# Global Proteomic Profiling of Cerebral Malaria-Infected Erythrocytes Reveals a Specific *Pf*EMP1 Expression Pattern

**DOI:** 10.1016/j.mcpro.2026.101659

**Published:** 2026-09-10

**Authors:** Jérémy Fraering, Virginie Salnot, Valentin Joste, Bertin Vianou, Nicolas Argy, Romain Coppée, Florence Migot-Nabias, Emilie-Fleur Gautier, Gwladys Irénée Bertin, NeuroCM study group

**Affiliations:** 1 UMR261 MERIT, Université Paris Cité, IRD, INSERM U1344, Paris, France; 2 Plateforme Proteom'IC, Institut Cochin, Université Paris Cité, INSERM U-1016, CNRS UMR8104, Paris, France; 3 Plateforme Protéomique Strasbourg Esplanade du CNRS, Université de Strasbourg, Strasbourg, France; 4 Université Paris Cité, INSERM, Biologie Intégrée du Globule Rouge, Paris, France; 5 Laboratoire Rate, Infections et Parasites, Institut Pasteur, Paris, France; 6 Laboratoire de Parasitologie, Hôpital Bichat-Claude Bernard, APHP, Paris, France; 7 Laboratoire de parasitologie-mycologie, UR 7510 ESCAPE, Université de Rouen Normandie, Rouen, France; 8 Initiatives IdEx Globule Rouge d'Excellence (InIdex GR-Ex), Université Paris Cité, Paris, France

**Keywords:** cerebral malaria, *P. falciparum*, DIA proteomic, blood, *Pf*EMP1

## Abstract

Cerebral malaria (CM) is the most lethal complication of malaria, causing severe impairment of brain function. Although the precise mechanisms leading to CM remain unknown, the adhesion of infected erythrocytes to the cerebral endothelium, mediated by *Plasmodium falciparum* erythrocyte membrane protein 1 (*Pf*EMP1) proteins, is considered a key factor. These proteins, encoded by *var* genes, are divided into several groups and contain specific domain cassettes (DCs) already known to be associated with CM. In this study, we compared the human and parasite proteomes of *Plasmodium falciparum*–infected erythrocytes from 19 samples from the NeuroCM cohort, consisting of 10 children with uncomplicated malaria and 9 with CM from Benin. We used data-independent acquisition mass spectrometry with trapped ion mobility spectrometry and parallel accumulation serial fragmentation coupled to recent *Pf*EMP1 databases to analyze our samples. Our results identified over 7000 proteins, including both host and parasite proteins. Analysis of the host proteome revealed an increased abundance of reticulocyte-specific proteins in CM cases, suggesting a possible parasite preference for invading erythroid precursors. On the parasite side, an increase in membrane proteins (PHIST, RIFIN, and STEVOR) was observed in CM cases. Moreover, *Pf*EMP1 proteins containing DC8 and DC13 were significantly more abundant in CM patients. These results provide protein-level confirmation of the association between DC8/DC13 of *Pf*EMP1 and CM, supporting the hypothesis of an enhanced parasite adhesion mechanism in CM. Our study highlights the importance of proteomic analysis in understanding the pathogenesis of CM and may contribute to the development of novel therapeutic strategies.

## Highlights

• CM reticulocyte markers rise, suggesting parasite tropism for erythroid precursors.
• PHIST, RIFIN, and STEVOR membrane proteins are enriched in CM.
• DC8/DC13 PfEMP1 variants rise in CM vs UM, supporting a role in CM pathogenesis.
• CIDRα1 enrichment in CM-enriched PfEMP1 supports a key role for EPCR-binding forms.

## Graphical Abstract

**Figure:**
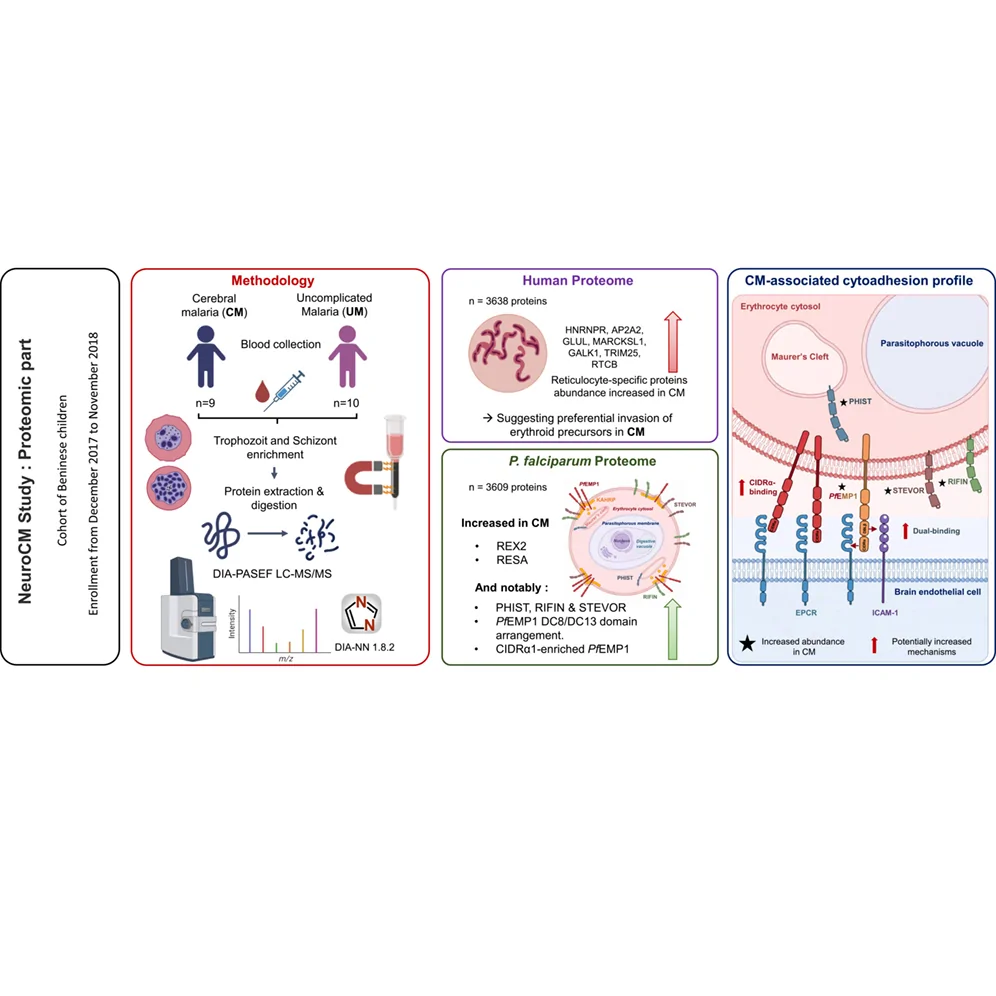

## In Brief

Using DIA-PASEF proteomics, we characterized the host and parasite proteomes of *Plasmodium falciparum*-infected erythrocytes from children with cerebral or uncomplicated malaria. Cerebral malaria (CM) was associated with increased abundance of reticulocyte-specific host proteins and parasite membrane proteins, including PHIST, RIFIN, and STEVOR. Importantly, *Pf*EMP1 proteins containing DC8 and DC13 were significantly more abundant in CM, providing protein-level evidence supporting their association with CM and enhanced cytoadherence.

Cerebral malaria (CM) is the most lethal complication of *Plasmodium falciparum* malaria, causing severe disturbances in brain function, including brain swelling, altered consciousness, and coma. It is fatal in most cases without treatment, and even with treatment, the mortality rate remains high (15–20%) (1). According to the World Health Organization (WHO), malaria caused 610,000 deaths in 2024 (https://www.who.int/teams/global-malaria-programme/reports/world-malaria-report-2025), with 95% in Africa and 76% in children under five, who have not fully developed immunity. It is estimated that 1% of uncomplicated malaria (UM) cases in Africa progress to severe cases (2, 3). The specific reasons for the development of CM remain unknown, but the best described pathophysiological mechanism is the adhesion of *P. falciparum*-infected erythrocytes to the cerebral vascular endothelium, mediated by variant surface antigens (VSAs).

Among these antigens, the *Plasmodium falciparum* erythrocyte membrane protein 1 (*Pf*EMP1) proteins have been the most studied. *Pf*EMP1 are variant surface proteins expressed on the surface of infected erythrocytes that mediate adhesion to host endothelial receptors, allowing sequestration in the microvasculature and contributing to immune evasion and disease severity. They are encoded by a hypervariable gene family called *var*, comprising approximately 60 genes per parasite genome, with one gene expressed at a time. This family is divided into 4 groups (UPSA, UPSB, UPSC, and UPSE) and 2 intermediate groups (UPSB/A and UPSB/C) based on their chromosomal location and direction of transcription (4). Numerous studies have shown that the expression levels of *Pf*EMP1 transcripts from groups A and B/A may be associated with CM (5, 6, 7, 8, 9, 10, 11). Some specific sequences from the cysteine-rich interdomain regions (CIDRs) and Duffy-binding-like (DBL) domains of *Pf*EMP1, also referred to as domain cassettes (DCs), appear to be associated with CM. Indeed, studies of phenotypically selected strains for adherence to endothelial cells as well as patient samples have shown preferential expression of DC8 (DBLα2-CIDRα1.1/8-DBLβ12-DBLγ4/6) and DC13 (DBLα1.7/2-CIDRα1.4) (10, 12, 13) suggesting a role in CM pathology. Specific domains have been shown to interact with host endothelial receptors through protein-protein binding, such as DBLβ1/3, which binds to the intercellular adhesion molecule 1 (ICAM-1) receptor and for which a binding motif (I[V/L]x3N[E]GG[P/A]xYx27GPPx3H) has been described (14), and CIDRα1, which binds to the endothelial protein C receptor (EPCR) (15, 16). The vast majority of studies focusing on parasite adhesion and *var* characterization have used transcriptomic approaches. However, it is known that mRNA does not accurately reflect the amount of protein expressed (17, 18). Very few studies have used proteomics to analyze clinical field samples. Thus, a proteomic approach could allow to be more confident about the expression of these *Pf*EMP1 in parasitized red blood cells (RBCs).

However, few studies have investigated this aspect because *Pf*EMP1 proteins are highly variable and thus differ significantly from the sequences reported in reference databases, making it very difficult to assign identified peptides to the *Pf*EMP1 proteins. Moreover, handling field samples is challenging, making it difficult to collect high-quality samples. Bertin *et al.* quantified more than 440 *Pf*EMP1 peptides by LC-MS/MS analysis of parasitized RBCs (13). They showed that DC8 peptides were more abundant in CM cases than in UM cases. Later, in 2018, Kamaliddin *et al.* identified a total of 57 *Pf*EMP1 proteins by proteogenomic analysis, of which only 10 were derived from referenced databases (19). Of these, 147 peptides were identified, including 22% of peptides from the DBLδ domain, 15% from CIDRβ, 14% from NTS, and 10% from ATS, indicating that these domains were the most abundant in field isolates in Benin by LC-MS/MS analysis. However, these studies did not allow quantitative differentiation of *Pf*EMP1 between UM and CM samples, mainly due to the low sensitivity of LC-MS/MS analysis and a large number of missing values.

In this study, we took advantage of several recent technological advances in mass spectrometry (timsTOF HT, data-independent acquisition (DIA), and parallel accumulation-serial fragmentation [PASEF]) as well as the use of data-independent acquisition by neural networks (DIA-NN) (20) to generate a spectral library of *Pf*EMP1 domains from the VarDom for laboratory strains (21) and Varia for field isolates (22) databases, which significantly improved the quantification of *Pf*EMP1 proteins by LC-MS/MS. We analyzed by mass spectrometry 19 samples (10 UM and 9 CM) of parasitized RBCs from children infected with *P. falciparum* from the NeuroCM cohort. The NeuroCM study aimed to better understand neuroinflammation and recovery in young children with cerebral or UM. It integrated field-based approaches (23), clinical data (24), and transcriptomic analyses (25, 26). In the present study, we complement these previous investigations with an in-depth LC-MS/MS-based proteomic analysis of *P. falciparum*-infected erythrocytes. We aimed to compare host and parasite proteomic profiles between UM and CM cases and to determine whether specific *Pf*EMP1 proteins or domains are preferentially abundant in CM, thereby providing protein-level support for previously reported associations between var/*Pf*EMP1 features and CM.

## Experimental Procedures

### NeuroCM Patient Recruitment

Patient enrollment for the NeuroCM study occurred in Cotonou, southern Benin, from December 2017 to November 2018 and were performed in accordance with the ethical principles of the Declaration of Helsinki. A total of 171 children aged 2 to 6 years were recruited (73 CM, 98 UM). The comprehensive clinical and biological parameters of these participants are detailed in previous publications by our group (23, 25). Inclusion criteria were based on World Health Organization guidelines (https://www.who.int/publications-detail-redirect/9789241548526). Briefly, CM was characterized by confirmed *P. falciparum* infection combined with a Blantyre Coma Score of less than 3, excluding patients with other causes of coma (23, 25). UM was defined by fever at enrollment or within 24 h, *P. falciparum* presence, and absence of severe malaria signs. Recruitment was conducted at two hospitals: the CHU de la Mère et de l’Enfant Lagune and the Hôpital de zone de Calavi. Routine laboratory tests for children with coma were performed locally, while those for children with UM were performed at the Institut de Recherche Clinique du Bénin.

### Patient Sample Preparation

The initial phase of sample preparation was performed at the laboratory of the Centre d'Etude et de Recherche sur les Pathologies Associées à la Grossesse et à l'Enfance. Between 1 and 1.5 ml of patients’ whole blood was cultured in RPMI 1640 medium supplemented with 0.5% Albumax (Gibco) for less than 48 h (23). This process allowed the parasites present in the circulating blood of the patients to mature *in vitro* into an older form of the parasite cycle (from young trophozoite to schizont). These mature parasites express the highest number of proteins. Following this step, mature parasites were sorted using a magnetic-activated cell sorting (MACS) technique (Miltenyi Biotech). The parasitemia of the purified post-MACS fraction of mature forms was calculated by microscopic observation to assess the yield of the purification and also to ensure a similar parasitemia among the samples to be analyzed by mass spectrometry, thus avoiding potential quantification bias due to a higher ratio of healthy to infected RBCs. These pellets of mature parasites were then stored at −80 °C and transported to Paris for proteomic analysis on the Protéom'IC platform.

### Sample Selection and Preparation for LC-MS/MS Analysis

Samples for analysis were selected based on several criteria. The success of MACS purification of mature forms was considered (post-MACS parasitemia >70%) to select 19 samples (9 CM and 10 UM) for LC-MS/MS analysis. We did not normalize the trophozoite/schizont ratio post-MACS, which may introduce variability unrelated to clinical differences. Pellets of parasitized RBCs were first lysed using SDS lysis buffer (2% SDS, 100 mM TEAB pH 8.5). The volume of lysis buffer used for each sample was estimated based on the total cell count observed under the microscope. The samples were then heated at 95 °C for 5 min, centrifuged, and the supernatants collected. A bicinchoninic acid assay (Thermo Fisher Scientific Pierce BCA Protein Assay Kit) was performed on the supernatants to estimate the total amount of protein in each sample. A volume corresponding to 40 μg of protein was then taken from each sample, diluted in reduction and alkylation buffer (final concentration: 2% SDS, 50 mM chloroacetamide, 10 mM tris(2-carboxyethyl)phosphine, 200 mM TEAB pH 8.5) and heated at 95 °C for 5 min. Samples were then acidified to pH < 2 with 10% (v/v) of 12% phosphoric acid, followed by the addition of six volumes of S-Trap binding buffer (90% MeOH/100 mM TEAB pH 8.5) before loading onto an S-Trap column (Protifi). After three washes with S-Trap binding buffer, 1 μg of trypsin was added in 100 μl of 50 mM TEAB buffer and applied to each column. The proteins in each sample were then digested overnight at 37 °C. The next day, the peptides were eluted in three consecutive steps (50 mM TEAB; 0.2% formic acid; 50% acetonitrile/0.2% formic acid) and the peptides were dried using a SpeedVac.

### Analysis of Parasitized RBCs by LC-MS/MS

Peptides were resuspended in 30 μl of 10% acetonitrile/0.1% TFA. Five microliters of peptides were injected into a Dionex U3000 nano-LC over a 4-h gradient coupled to a timsTOF HT mass spectrometer (Bruker). A DIA method with active PASEF was established using Data Analysis and timsControl software (Bruker). Samples were randomly injected into the mass spectrometer. On the nLC, peptides were washed on a reversed phase C18 trap column (5μm particle size, 300 μm inner diameter, 5 mm length, from Thermo Fisher Scientific) and separated on a reversed-phase C18 column (1.6 μm particle size Aurora, 75 μm inner diameter and 25 cm length from IonOptics), then injected into the mass spectrometer by electrospray. For the DIA-PASEF MS method (64 nonoverlapping windows), the ion-mobility range was 0.45 to 1.42 1/K_0_ and the mass range 400–1200 Da (See supplemental methods for exact DIA-windows). We used 12 cycles, yielding an estimated total cycle time of 1.38 s. Accumulation and ramp times were set to 100 ms.

### Search Parameters

The raw data obtained from the mass spectrometer were analyzed using DIA-NN version 1.8.2 software (20). Spectral libraries were built in library-free mode assuming trypsin cleavage (one miss cleavage) and specifying carbamidomethyl (C) as fixed and oxidation (M) and N-terminal acetylation as variable modifications. Three independent analyses were performed with mass tolerance set to 0 in DIA-NN (optimized tolerance window through the first file-see pride data report.log). All three analyses included human proteins (UniProt reference proteome release 2023_04 which contains 42,382 Swiss-Prot – with isoforms – and 61,448 Trembl entries) and proteins from the 3D7 strain of *P. falciparum* (PlasmoDB v54 for VarDom and v68 for Varia both containing the same amount of entries: 5389). In addition, we incorporated (DIA-NN analysis A) the *Pf*EMP1 protein database from VarDom (21) (v1.1 – containing 403 full-length protein entries), or (DIA-NN analysis B) the *Pf*EMP1 domains database from VarDom (v1.1 - containing 2477 domains), and (DIA-NN analysis C) the Varia (containing 1,064,250 entries) database (22). Protein identification was performed with a false discovery rate (FDR) threshold of 1% (at both precursor and protein levels in all libraries), and match between runs was enabled. Protein quantification and intensity normalization were performed by DIA-NN using the MaxLFQ algorithm. The output data from DIA-NN were then analyzed using R and Perseus (27). DIA-NN-normalized Label-Free Quantification (LFQ) values were transformed to Log2(x). For the global proteome, R software was used to generate descriptive data (https://github.com/MarjorieLeduc/PROXIMA). Proteins were retained for statistical analysis based on reliable quantification data, using a minimum threshold of 70% valid values in at least one group for human and *P. falciparum* proteins, and a 40% threshold for Varia proteins.

### Functional Analysis

Functional analysis was performed using the STRING online tool. The “Proteins with values/rank” tool was used for global proteome enrichment analysis by providing the gene name and associated Log2FoldChange value for each protein. The “Multiple proteins” tool was used for pathway analysis of significantly abundant proteins.

### Experimental Design and Statistical Rationale

A subset corresponding to approximately 10% of the total cohort (n = 19) was analyzed as biological replicates and no technical replicates were performed. Mann-Whitney U-tests were performed for clinical and biological parameters, except for sex ratio, for which a chi-squared test was performed. Student's *t*-tests were performed to compare protein abundance levels between samples from UM and CM patients. For the human proteome, a Benjamini-Hochberg correction for multiple testing was applied, and proteins with a *q*-value <0.05 were considered significantly deregulated. Given the high rate of missing values in the parasite and *var* proteomes, we decided to select significantly deregulated proteins with a threshold of *p* value <0.01 for all parasitic proteins and a threshold of *p* value <0.05 for the focus on *Pf*EMP1 proteins without correction with a fold change < −0.5 or >0.5. It is important to note that these latter tests, without Benjamini-Hochberg corrections, may result in a higher probability of false positives and therefore the interpretation of these results should be approached with caution.

Correlation analyses were performed in R using Spearman’s rank correlation test. For each pair of variables, only complete observations were retained, and missing values were excluded pairwise. Spearman’s correlation coefficient (ρ) and the associated *p* value were calculated using the cor.test() function from base R with method = "spearman" and exact = FALSE. To account for multiple testing, *p* values were adjusted using the Benjamini-Hochberg FDR procedure with the R function p.adjust() and method = "BH". Correlations were considered statistically significant according to the selected alpha threshold using either raw *p* values or FDR-adjusted q-values, as indicated.

### Study Approval

Ethical approval was obtained from the Comité National d’Ethique pour la recherche en santé au Benin (N°67/MS/DC/SGM/DRFMT/CNERS/SA; October 17, 2017) and the Comité consultatif de déontologie et d’éthique of the Institut de Recherche pour le Développement (IRD; October 24, 2017).

## Results

### Clinical and Biological Description of Samples Included in the LC-MS/MS Analysis

A total of 19 samples of parasitized RBCs from children with UM (n = 10) and CM (n = 9) were analyzed. The clinical and biological data for this subset of patients, associated with the binding phenotype of clinical *P. falciparum* isolates on HBEC-5i cells and CHO-ICAM-1 cells (25), are detailed in Table 1. In this subset, it was observed that children with UM (53 months) were significantly older (*p* = 0.0172, Mann-Whitney U-test) than those with CM (44 months). Furthermore, parasite densities and leukocyte counts were significantly higher (*p* = 0.0288) in CM cases (576,470 parasites/μl and 17,830 leukocytes/μl, respectively) compared to UM cases (184,434 parasites/μl and 9990 leukocytes/μl, respectively). Adherence data in this subset showed significantly greater parasite adherence to HBEC-5i and CHO ICAM-1 cells (*p* = 1.150^E-02^ and *p* = 6.993^E-03^, respectively) for CM samples (76.29 IE/mm^2^ and 162.88 IE/mm^2^, respectively) compared to UM samples (28.11 IE/mm^2^ and 47.00 IE/mm^2^, respectively). Children with CM had significantly more severe anemia than those with UM (*p* = 3.725^E-02^), with hemoglobin levels of 6.42 g/dl in CM and 9.15 g/dl in UM.

**Table 1:** Clinical and biological characteristics of included patients

Parameters	Cerebral malaria, mean [SD] (n = 10)	Uncomplicated malaria, mean [SD] (n = 10)	*P* value
Parasite density (parasite/μl)	576,470 [499,207]	184,434 [8,739]	**2.88E-02**
Age (month)	43.74 [11.02]	53.05 [8.64]	**1.72E-02**
Sex (% female)	50%	60%	1.00E+00
Blantyre score	2 [0.42]	5 [0]	**3.29E-05**
Hemoglobin (g/dl)	6.42 [1.91]	9.15 [1.49]	**3.73E-02**
Leucocytes (10ˆ3/μl)	17.83 [10.23]	9.99 [7.75]	**2.32E-02**
Blood glucose (g/L)	0.607 [0.55]	0.98 [0.31]	1.40E-01
Creatinine	4.37 [1.73]	3.56 [0.74]	3.08E-01
Mean corpuscular volume	70.8 [6.71]	69.1 [9.72]	5.95E-01
Adherence Hbec-5i (IE/mm^2^)	76.29 [61.34]	28.11 [25.92]	**1.15E-02**
Adherence CHO-ICAM1 (IE/mm^2^)	162.88 [86.19]	47.00 [31.08]	**6.99E-03**

The table summarizes data from 20 enrolled patients (10 with cerebral malaria and 10 with uncomplicated malaria). A Mann–Whitney U-test was performed to compare the groups, and characteristics that differed significantly are indicated by bold *p* values. IE = infected erythrocyte.

### Global Proteome Analysis of the Parasitized Red Blood Cells

First, the quantitative data from the CM10 sample were excluded from the final analysis due to a significantly higher contamination with white blood cell proteins compared to the other samples, which biased the overall quantification data (Supplemental Fig. S1). As a result, nine CM and 10 UM samples were analyzed. A total of 7392 proteins were identified (DIA-NN analysis B), of which 3638 were human proteins (Uniprot), 3609 were parasite proteins (PlasmoDB), and 145 were *Pf*EMP1 domains (VarDom). Approximately less than 6000 different proteins were identified per sample, with less than 25% missing values (Supplemental Fig. S2*A*). Pearson’s correlation coefficients are high (between 0.84 and 1) for all comparisons (Supplemental Fig. S2*B*). However, higher Pearson’s coefficients were found in the UM group indicating greater homogeneity within this group compared to the CM group. Principal component analysis (PCA) on the raw LFQ data of the entire dataset partially separate the clinical groups based on Component 1, which explained 29.4% of the variance (Supplemental Fig. S2*C*). Overall, these results demonstrate high-quality data essential for a meaningful differential expression analysis.

### Host Proteome Analysis of the Parasitized RBCs

In this initial section, we aimed to examine how the host proteome differs between UM and CM. Among the 3638 human proteins identified (DIA-NN analysis B), 2257 passed the valid-value filter and were retained for differential analysis; 74 were quantified exclusively in UM and 181 in CM (Fig. 1*A*). The PCA revealed a separation between CM and UM along Component 2, accounting for 10.3% of variability except for UM6 and CM6 (Fig. 1*B*). We also showed that CM2, CM5, CM6, and CM9 seem to cluster together, apart from the other CMs. Global enrichment analysis of these proteins revealed significant enrichment of four lipid oxidation pathways (each with ∼15 proteins): fatty acid beta-oxidation (GO:0006635), fatty acid catabolic process (GO:0009062), fatty acid oxidation (GO:0019395), and lipid oxidation (GO:0034440), with all associated proteins being more abundant in CM compared to UM (Supplemental Data 1). Four additional pathways (each with ∼150 proteins) involved in peptide modification or proteasomal degradation were significantly enriched: protein modification by small protein conjugation or removal (GO:0070647), ubiquitin-dependent protein catabolic process (GO:0006511), modification-dependent macromolecule catabolic process (GO:0043632), and modification-dependent protein catabolic process (GO:0019941). Within these pathways, fewer than 10 proteins were less abundant in CM (log2FC < −0.3); approximately 80 proteins had log2FC values close to zero (−0.3 to 0.3), and 60 had values > 0.3, indicating a trend toward higher abundance in CM patients. Differential protein abundance analysis identified 61 significantly deregulated proteins (*q*-value <0.05; Fig. 1*C* and Supplemental Data 2). All were more abundant in CM except tubulin alpha-8, which was more abundant in UM (log2FC = −1.20; *q*-val = 4.22E-02). Functional analysis revealed overrepresentation of proteins involved in clathrin-dependent endocytosis (GO:0072583) including CLTB, CLTA, AP2A2, and AP2S1 and in metabolite and energy production (GO:0006091) including GAPDH, BID, CYCS, PGK1, CAT, TALDO1, GALK1, GNPDA1, and ETFB as well as various intracellular transport pathways (GO:0046907; GO:0071705; GO:0071702; GO:0006886; Supplemental Data 3). Notably, seven proteins specifically found in reticulocytes (28) (HNRNPR, AP2A2, GLUL, MARCKSL1, GALK1, TRIM25, RTCB) were more abundant in CM (log2FC = 1.34; 1.00; 0.89; 1.02; 0.75; 0.98; 1.22, respectively). In addition, the reticulocyte marker transferrin receptor (TFRC/CD71) showed a relatively high log2FC (0.70) but was not statistically significant.

**Fig. 1:**
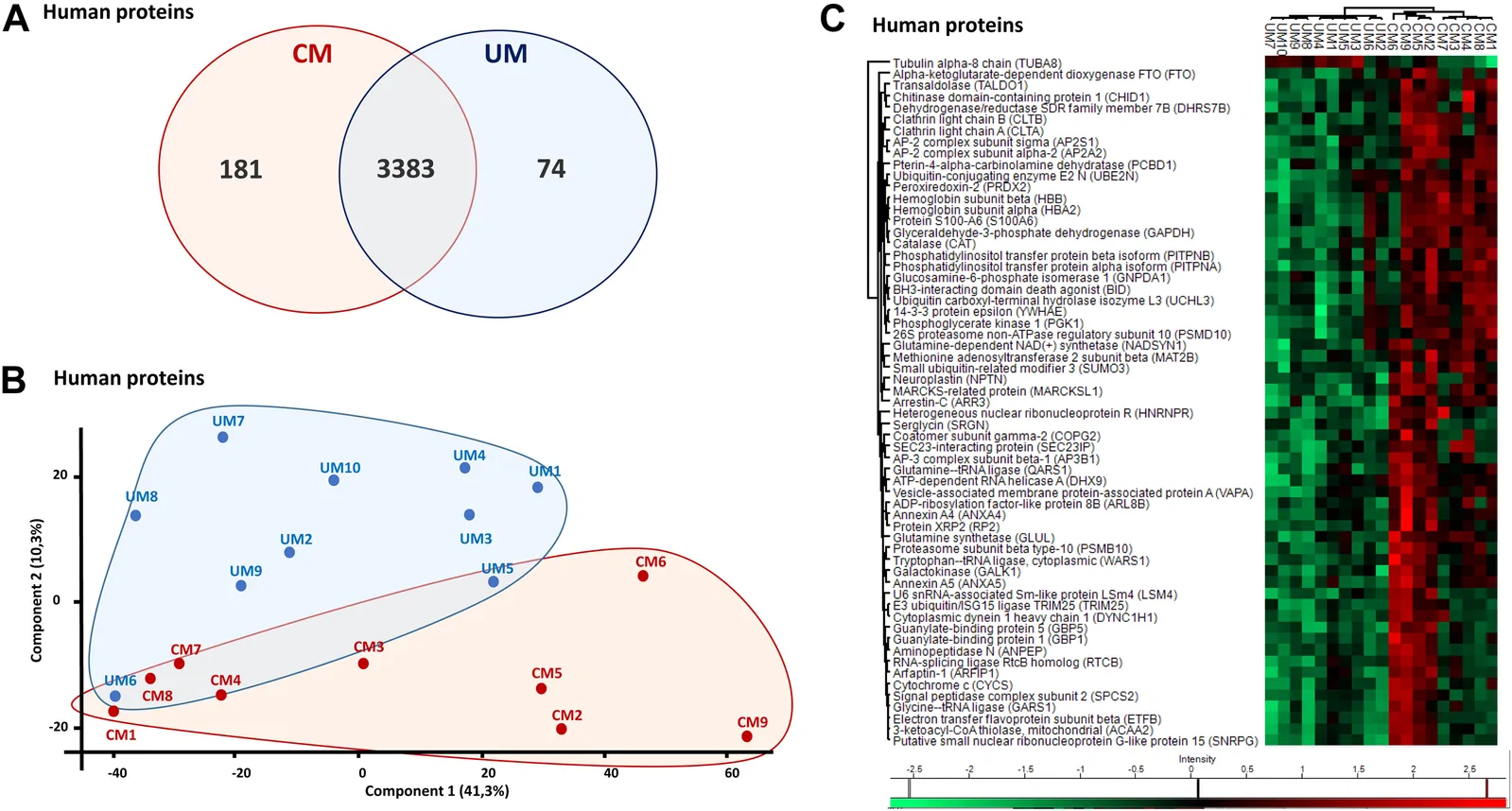
**Human proteome analysis.***A*, Venn diagram of the human protein identified in *P. falciparum*-infected erythrocyte from NeuroCM cohort (UM in *blue*; CM in *red*). *B*, principal component analysis (PCA) of the human protein quantified by LC-MS/MS (UM in *blue*; CM in *red*). *C*, hierarchical clustering (Pearson correlation) made with Perseus software of the differentially abundant human proteins (Student *t* test with Benjamini-Hochberg correction with a *q*-value <0.05), and after statistical analysis missing values were imputed according to Gaussian distribution with Perseus tool only for data visualization. CM, cerebral malaria; UM, uncomplicated malaria.

### Parasite Proteome Analysis of the Parasitized Red Blood Cells

In total we quantified 3609 parasite proteins (DIA-NN analysis B) in all our samples (Supplemental Fig. S3*A*), and 3234 proteins were retained. The PCA did not show a real group separation between clinical presentations, only CM1 and CM8 seemed to be distinct from all others (Supplemental Fig. S3*B*). We found 137 differentially abundant proteins (Supplemental Data 4), and no biological pathways seemed to be overrepresented among these differential proteins. However, we identified 13 proteins located in Maurer's clefts that were significantly more abundant in CM isolates than in UM, including four PHIST proteins from b and c groups (PF3D7_1476200; PF3D7_0830600; PF3D7_0219700; and unclassified PF3D7_0935700). RIFIN (PF3D7_0222600), ring-exported protein 2 (REX2 - PF3D7_0936000), ring-infected erythrocyte surface antigen (RESA - PF3D7_1149200) and STEVOR (PF3D7_1479500) that carried also high log2FC values (0.90; 0.63; 0.71; 2.20, respectively).

As a second step, we also adjusted the LFQ values according to the estimated trophozoite/schizont proportion of each sample, which was calculated using five markers for each parasite stage population (see Supplemental Methods). For the global parasite proteome, this stage adjustment appeared to affect the statistical analysis. Some proteins that did not reach significance after multiple-testing correction before stage adjustment became significant after adjustment. This was notably the case for STEVOR (PF3D7_1479500) and one PHISTb protein (PF3D7_1476200), which were significantly more abundant in CM samples (q-value <0.05), with high log2FC values of 2.28168 and 1.10804, respectively (Supplemental Data 5). For these two proteins, the abundance ratios did not appear to be strongly affected by the correction. This was also consistent with the PCA performed before and after normalization according to parasite stage proportion (Supplemental Fig. S4), suggesting that stage-based normalization did not drastically modify the overall dataset. In addition, a larger number of proteins reached significance at *p* value <0.01 after stage adjustment (Supplemental Data 5).

### *Pf*EMP1 Protein and Domain Investigation by LC-MS/MS in the Context of CM

Next, we turned our attention to the examination of *Pf*EMP1 expression patterns, aiming to gain deeper insights into the parasite’s pathogenic mechanisms. Following the DIA-NN analysis A (See the Materials and Methods section), a total of 89 *Pf*EMP1 proteins from the VarDom database, representing seven *P. falciparum* laboratory strain proteomes (21), were quantified across all our isolates, but 2 of them were in protein groups and cannot be discriminated. Of these 87 remaining proteins, 43 belonged to *Pf*EMP1s group B, 23 to group A, 7 to group C, 1 to group E and 13 proteins were not assigned to any group (Fig. 2*A* – Supplemental Data 6). Differential protein abundance analysis revealed that nine *Pf*EMP1 proteins were significantly deregulated (*p* < 0.05, Student’s *t* test). The LFQ z-scored intensities of these nine proteins are shown on the heatmap in Figure 2*B*. Notably, only one of them, HB3var05, which contains a DC5 arrangement (DBLγ12-DBLδ5-CIDRβ3-DBLβ7) (Fig. 2*C*), was found to be more abundant in UM than in CM (Log2FC = −1.79; *p* = 0.00998), while the others were significantly more abundant in CM. The majority of the differentially regulated proteins belonged to the UPSA group (6 out of 9), two were assigned to the UPSB group, and one remained undetermined (Fig. 2*C*). Notably, six *Pf*EMP1 proteins contained a CIDRα1 domain. In addition, AAQ73928 (Log2FC = 0.96; *p* = 4.63^E-02^) from the UPSA group had a DC13 arrangement (DBLα1.7-CIDRα1.4) and IT4var06 (Log2FC = 1.81; *p* = 2.58^E-02^) from the UPSB group had a DC8 arrangement (DBLα2-CIDRα1.1-DBLβ12-DBLγ6) in their respective protein sequences (Fig. 2*C*) and were both more abundant in CM than in UM isolates. These data were also examined at the peptide-abundance level using the unique peptides assigned to each deregulated *Pf*EMP1 protein. Overall, the peptide-level data confirmed the same abundance trends, except for HB3var1csa, DD2var40, and AAQ73928 (Table 2) in which some peptides did not follow the trends. In addition, we also assessed the effect of stage-proportion adjustment on the *Pf*EMP1 analysis. However, this adjustment appeared to have a negative impact on the statistical analysis of *Pf*EMP1 proteins (Supplemental Data 6b). Indeed, *Pf*EMP1 quantification is characterized by a high proportion of missing values. In this context, even moderate modifications of LFQ values during stage adjustment are more likely to substantially alter the statistical results. Therefore, the stage-adjusted *Pf*EMP1 results should be interpreted with caution.

**Fig. 2:**
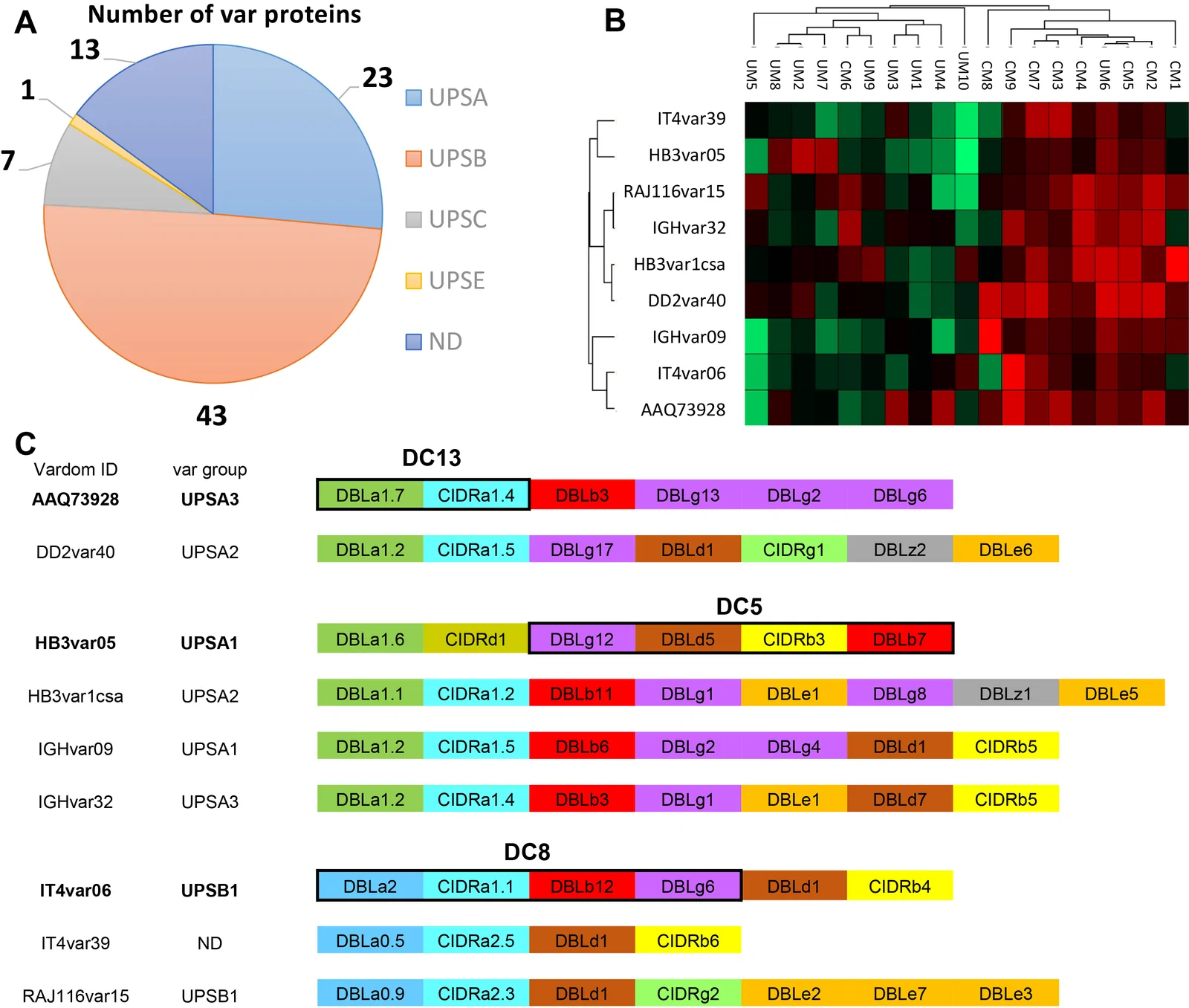
***Pf*EMP1 Analysis.***A*, diagram representing the number of *Pf*EMP1 of each family identified by LC-MS/MS by querying VarDom database. *B*, hierarchical clustering (Pearson correlation) made with Perseus software of the differentially abundant *Pf*EMP1 observed between CM and UM presentation (Student’s *t* test with a *p* value <0.05), and after statistical analysis missing values were imputed according to Gaussian distribution with Perseus tool only for data visualization. *C*, domain composition of deregulated *Pf*EMP1 proteins (source: VarDom) in CM with the known domain cassette outlined in *black*. CM, cerebral malaria; PfEMP1, *Plasmodium falciparum* erythrocyte membrane protein 1; UM, uncomplicated malaria.

**Table 2:** Comparison of *Pf*EMP1 abundance between unique peptides and protein quantification

Protein.Group	Stripped.Sequence	Precursor.Charge	Unique peptide level log2FoldChange (CM/UM)	Protein level log2Foldchange (CM/UM)	Tendency
IT4var39	VENGEHK	2	1,462	1,388	ok
HB3var05	DVITILLNR	2	−1,827	−1,794	ok
RAJ116var15	KEYDFQK	2	0,367	0,710	ok
RAJ116var15	LNDESICK	2	Only CM	0,710	ok
IGHvar32	LKDGFIK	2	0,698	0,644	ok
IGHvar32	WNNKEDILNK	2	0,514	0,644	ok
HB3var1csa	MGGYLPPLEIK	2	Only UM	1,171	not ok
DD2var40	AHVWHAMLCGYRK	2	−1,308	1,233	not ok
DD2var40	AHVWHAMLCGYRK	3	0,694	1,233	ok
DD2var40	ILEESQYK	2	−0,285	1,233	not ok
DD2var40	SGNSPTNTNNTTTSHDNMGEKPFITSIHDR	4	Only UM	1,233	not ok
DD2var40	TYKNSVNK	2	Only CM	1,233	ok
IGHvar09	CSNECLGYENWIK	2	Only CM	1,689	ok
IGHvar09	ENYPDWDCIK	2	Only CM	1,689	ok
IGHvar09	LLIDTINWEYK	2	Only CM	1,689	ok
IGHvar09	NDKVENGLK	2	1,042	1,689	ok
IGHvar09	SFLNLLNEGK	2	2,984	1,689	ok
IT4var06	YEGESLVEK	2	1,269	1,811	ok
IT4var06	YSFADIADIIR	2	Only CM	1,811	ok
AAQ73928	AHVQLEER	2	2,265	0,962	ok
AAQ73928	IVDGILTGK	2	Only UM	0,962	not ok
AAQ73928	KAHVQLEER	2	−1,160	0,962	not ok
AAQ73928	SLNPCHGK	2	Only CM	0,962	ok

The table reports the fold-change values between UM and CM from the standard protein-level abundance analysis, together with the corresponding abundance data for unique peptides from the nine *Pf*EMP1 proteins found to be significantly deregulated in patients. Consistent trends between peptide- and protein-level abundance are displayed in tendency column.

Following the DIA-NN analysis B, the raw data were then queried against a spectral database containing the *Pf*EMP1 domains separately, rather than the entire proteins, keeping both human and parasite database. In this way, 145 *Pf*EMP1 domains were quantified in our dataset with more missing values than in the global human and parasite proteomes. For differential abundance, proteins with at least two values in at least one group were selected (n = 85). Four *Pf*EMP1 domains were found to be significantly more abundant in CM (Supplemental Data 6): NTSA7 (Log2FC = 1.57; *p* = 9.19^E-04^); DBLα1.2 (Log2FC = 1.61; *p* = 3.98^E-02^); CIDRα1.1 (Log2FC = 1.55; *p* = 3.59^E-02^) and DBLδ1 (Log2FC = 1.13; *p* = 4.60^E-02^).

We then queried our data using the Varia database (22). Following the DIA-NN analysis C, we identified over 649 *Pf*EMP1 domain sequences, noting that some domains are repeated across multiple *Pf*EMP1. This represents an increase of more than 500 sequences compared to the results obtained with VarDom (DIA-NN analyis B). Of the 649 *var* domain sequences, 243 domains were initially retained after filtering out proteins with less than 40% of valid values in at least one group. Twenty-six domains were found to be significantly deregulated in CM (*p* < 0.05 and Log2FC > 0.5 or < −0.5) (Supplemental Data 7). Among these deregulated domains, only DBLγ12 (Log2FC = −0.89) was found to be more abundant in UM, while all others were more abundant in CM. The most differentially expressed domains were CIDRβ1 (Log2FC = 1.82), DBLβ13 (Log2FC = 1.78), and ATS1A (Log2FC = 1.62). Of note, the NTSA7 (Log2FC = 1.50), DBLβ3 (Log2FC = 0.94), and DBLδ1 (Log2FC = 0.81) domains were also present.

Due to the large number of identical *var* domains present on different *Pf*EMP1 proteins, we also chose to average protein abundance by domain, primarily to attempt a comparison with the VarDom database. As a result, 134 distinct *Pf*EMP1 domains were identified using the Varia database (Supplemental Data 8). In this way, 14 domains were found to be significantly deregulated. CIDRd2 was the only domain found to be more abundant in UM (Log2FC = −1.70; *p* = 0.0260). NTSA7 and NTSB3 (*p* = 0.001 and 0.035, respectively) were more abundant in CM (Log2FC = 1.09 and 0.785, respectively), as was the variable var_all (average abundance of all ATS and NTS domains) with a small fold change (Log2FC = 0.35; *p* = 0.0036). Interestingly, five DBLa0 domains (DBLa0.11/.5/.8/.9/.4) were found to be more abundant in CM. For the same dataset, we observed approximately four times more peptides with the Varia database (n = 4342) compared to VarDom (n = 1170) (Fig. 3). There were 29 *Pf*EMP1 domains found exclusively in Varia (Supplemental Data 9), including DBLβ9 that got 61 identified precursors with Varia. Among the domains common to both databases, only 16 had more peptides with VarDom, including DBLg11 (n = 14), DBLg7 (n = 13), and DBLa1.7 (n = 11), which were the three with the highest difference. All others (n = 97) had more peptides identified with Varia with a significant increase in the number of peptides (Fig. 3 - Supplemental Data 10).

**Fig. 3:**
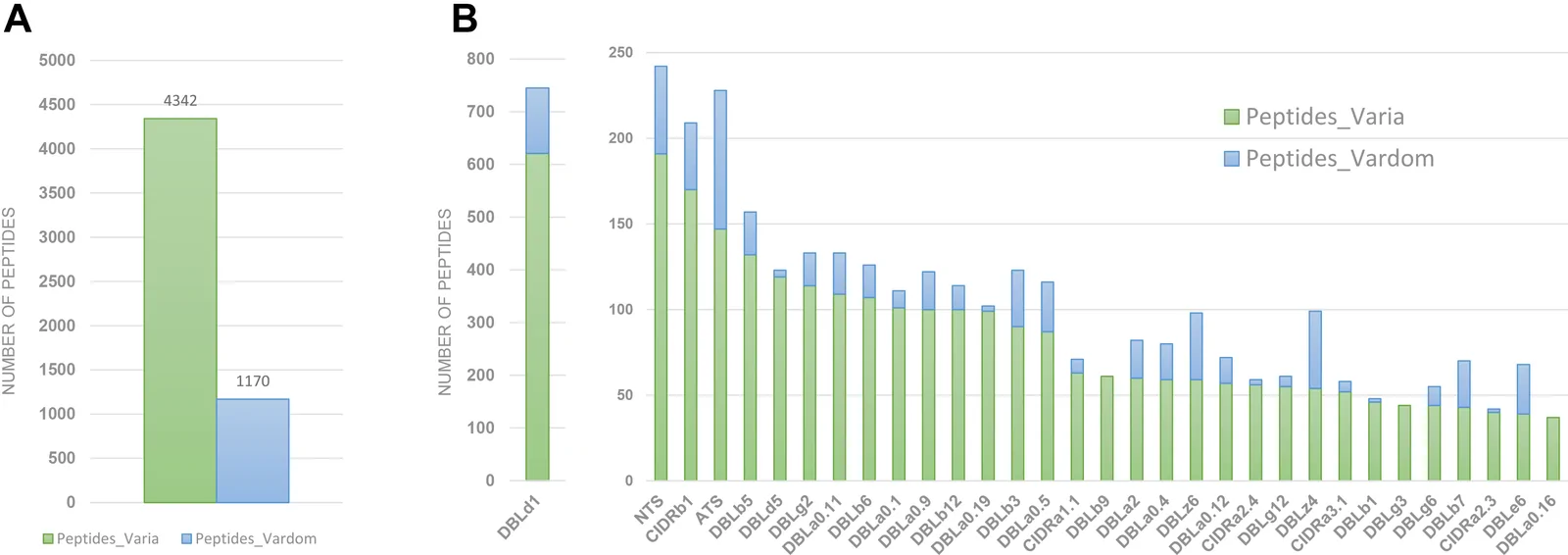
**Varia *versus* VarDom peptides analysis.***A*, histogram representing the total number of precursors identified by querying Varia (*green*) or VarDom (*blue*) in all samples. *B*, histogram representing the number of precursors identified with Varia (*green*) or VarDom (*blue*) in the Top31 of *var* domains quantified among all samples.

### Correlation Analysis Between Mass Spectrometry and Parasite Adhesion to Endothelial Cells

Spearman correlation analyses were performed to assess the relationship between *Pf*EMP1 quantification and parasite cytoadherence in samples from the same patients previously described in a previous study by our group (25). Overall, both domain-level expression (Fig. 4*A*) and full-length protein abundance (Fig. 4*B*) showed predominantly positive correlations with parasite adhesion. NTSA7 was the only domain whose correlation with adhesion to HBEC-5i cells remained significant after FDR correction (q-value = 0.019). This result is of particular interest because NTSA7 was detected in all samples, providing quantitative LC-MS/MS values for n = 19 samples. Moreover, NTSA7 is a relatively conserved domain across *Pf*EMP1 proteins, and may therefore serve as a proxy for the total *Pf*EMP1 abundance in our samples.

**Fig. 4:**
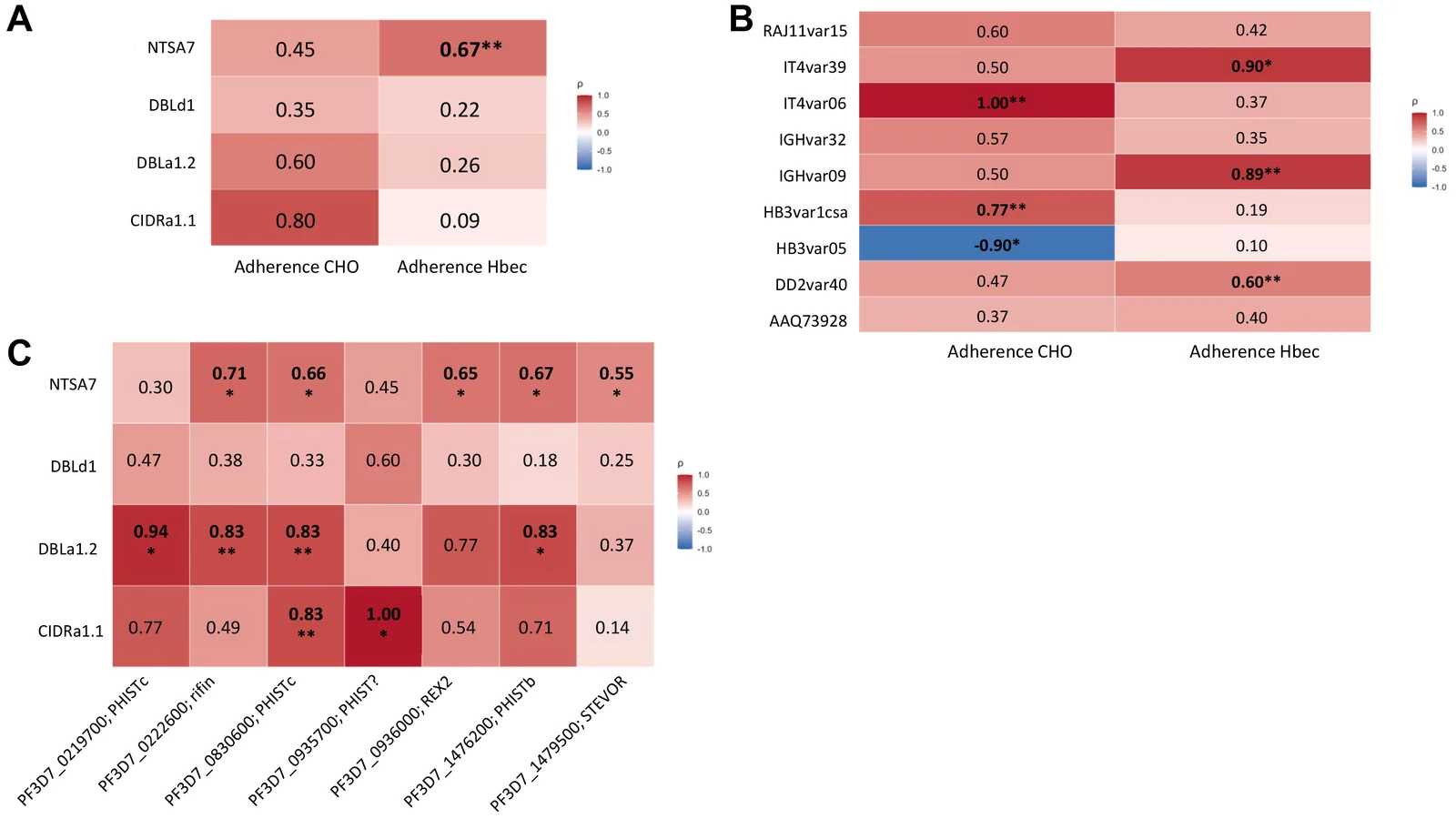
**Correlation analysis between *Pf*EMP1 abundance and parasite adhesion in samples from the same patients.***A*, heatmap showing Spearman correlations between the abundance of the four var domains found to be significantly more abundant in CM than in UM and parasite adhesion to HBEC-5i and CHO-ICAM-1 cells (∗*p*_val < 0.05; ∗∗q_val < 0.05). *B*, heatmap showing Spearman correlations between the abundance of the nine full-length *Pf*EMP1 proteins differentially abundant in CM and parasite adhesion to HBEC-5i and CHO-ICAM-1 cells (∗*p*_val < 0.05; ∗∗q_val < 0.05). *C*, heatmap showing Spearman correlations between *Pf*EMP1 domain abundance and the abundance of various parasite proteins quantified by LC-MS/MS and overexpressed in CM (∗*p*_val < 0.05; ∗∗q_val < 0.05). CHO, Chinese hamster ovary; CM, cerebral malaria; HBEC-5i, human brain endothelial cell line-5i; ICAM-1, intercellular adhesion molecule 1; PfEMP1, *Plasmodium falciparum* erythrocyte membrane protein 1; UM, uncomplicated malaria.

At the full-length *Pf*EMP1 protein level, IT4var39, IGHvar09, and DD2var40 showed significant positive correlations with parasite adhesion (*p* value = 0.037; q-value = 0.040; q-value = 0.040, respectively). These three proteins share the DBLδ1 domain, which was also the domain for which the highest number of peptides was identified (Fig. 3). However, unique peptides allowed these three proteins to be clearly distinguished in our analyses. HB3var05, in contrast, showed a negative correlation on CHO-ICAM-1 cells, suggesting that higher abundance of this *Pf*EMP1 protein may be associated with reduced parasite adhesion on ICAM-1 receptors. This observation appears consistent with the fact that HB3var05 was found more abundant in UM than in CM by LC-MSMS. Thus, this *Pf*EMP1 that contains a DC5 DC could potentially have a protective effect against CM.

We also correlated the LC-MS/MS-based abundance of several parasite proteins, including PHIST, STEVOR, and RIFIN proteins, with the abundance of *Pf*EMP1 domains (Fig. 4*C*). Overall, these results showed that most *Pf*EMP1 domains were positively correlated with the abundance of these parasite proteins, which are involved in *Pf*EMP1 trafficking. More interestingly, the NTSA7 and DBLα1.2 domains showed significant correlations with five and four of these parasite proteins, respectively, whereas no significant correlation was observed for DBLδ1.

## Discussion

To improve our understanding of CM pathophysiology in children, we performed untargeted LC-MS/MS analysis using DIA on 19 parasitized RBCs samples from pediatric cases of CM or UM. This approach resulted in the identification of over 7392 proteins, including 3638 human, 3609 parasite; and 145 *Pf*EMP1 domains (corresponding to 89 full-length proteins). The use of DIA significantly increased protein identification capacity and reduced missing values, strengthening statistical analyses compared to our previous study (29).

Our findings revealed a significant enrichment of host lipid oxidation pathways in CM cases. Oxidative stress induced by reactive oxygen species, mainly produced by neutrophils and heme release in malaria (30), is a key component of immune defense against pathogens (31). Increased lipid peroxidation affects both parasitized and healthy RBC membranes, leading to increased erythrocyte membrane rigidity and correlating with increased mortality in acute malaria infection (32, 33, 34). In addition, proteasome-related degradation pathways were significantly enriched, predominantly involving proteins more abundant in CM cases, suggesting increased proteolytic activity in CM. Among the 61 significantly deregulated host proteins, four involved in endocytosis (CLTB, CLTA, AP2A2, and AP2S1) were more abundant in CM isolates. While some studies have reported endocytic activity in mature RBCs, this remains controversial because mature erythrocytes typically lack endocytic machinery and other organelles (35, 36, 37). We also identified seven reticulocyte-specific proteins (28) which were significantly more abundant in CM than in UM. A recent *in vitro* study reported preferential invasion of reticulocytes by the *P. falciparum* laboratory strains 3D7 and Dd2 (38). Knowing that, these results may indicate a potential preference for immature erythrocyte invasion in CM. These observations also raise the hypothesis that disease severity in malaria may be linked to the extent of immature erythrocyte infection. However, a limitation of our study is the analysis of MACS-purified parasitized RBCs, which contain approximately 15 to 20% healthy RBCs, with variable reticulocyte percentages influenced by anemia. There is no consensus on the percentage of circulating reticulocytes in malaria anemia between CM and UM, and reports vary. As we lack reticulocyte count in our study, mean corpuscular volume showed no significant difference between UM (69.1 [9.72] fL) and CM (70.8 [6.71] fL), suggesting similar reticulocyte counts in our analyzed samples. Moreover, we did not confirm that the transferrin receptor (CD71 or TFRC) was significantly more abundant in CM than in UM, despite a relatively high fold change favoring CM, contrasting with our previous study (29).

Exploratory analysis of parasite proteins (see Supplemental Data 4, 4b, and 5) revealed an increased abundance of membrane-associated proteins in parasitized RBCs from CM cases, particularly four PHIST proteins, including PF3D7_1476200, which remained significant after multiple-testing correction following parasite stage normalization (Supplemental Data 4b), one RIFIN, and one STEVOR (PF3D7_1479500). Interestingly, the abundance of these latter proteins positively correlated with that of the four *Pf*EMP1 domains found to be significantly enriched in CM cases, particularly NTSA7 and DBLα1.2, two domains containing sequences conserved across most *Pf*EMP1 variants. These observations could potentially suggest that these parasite proteins could be involved in supporting *Pf*EMP1 expression or trafficking at the cell surface since some of them carried a PEXEL domain (39). The exact role of PHIST proteins is not fully understood; they may mediate modification of infected RBCs through interaction with band 4.1R protein, contribute to stabilization of exported proteins within the parasitized cell, and facilitate trafficking of proteins to the cell membrane, including *Pf*EMP1 (40, 41). The elevated abundance of these proteins in CM samples could potentially imply heightened RBC rigidity and increased *Pf*EMP1 export to the cell surface. Such mechanisms may account for the observed higher *in vitro* adherence of parasitized RBCs in CM compared to UM cases. RIFINs and STEVORs, which like *Pf*EMP1 are members of the VSA family, also showed elevated levels in CM isolates, supporting the notion of an enhanced adherence mechanism in CM. STEVOR proteins have been identified as contributors to rosetting through their interaction with glycophorin C and in immune evasion strategies (42). This evidence suggests that increased rosetting may be an important factor in CM pathogenesis.

Further analysis using the *Pf*EMP1 laboratory strain database (VarDom) revealed that eight proteins were significantly more abundant in parasite isolates from CM patients compared to UM. Two of these *Pf*EMP1s contain DCs (AAQ73928—DC13 and IT4var06—DC8) previously identified as associated with severe malaria, including CM (10, 12, 13). This suggests a specific *Pf*EMP1 abundance that correlates with infection severity, as previously described in transcriptomic analyses (12). Surprisingly, we observed a *Pf*EMP1 more abundant in UM cases (HB3var05), which possesses a DC5 arrangement (DBLγ12-DBLδ5-CIDRβ3-DBLβ7). The DC5 arrangement is less well understood; some studies have hypothesized its association with severe malaria (10, 43), but this was not supported at the protein level in our study. Moreover, the abundance of this protein was negatively correlated with adhesion to CHO-ICAM-1 cells, suggesting that this *Pf*EMP1 may be associated with reduced ICAM-1-mediated adhesion. However, another study showed that DC5-containing *Pf*EMP1 have been associated with PECAM-1-mediated cytoadhesion and also that antibodies against DC5 are acquired early in life and that individuals with high levels of these antibodies are less likely to develop febrile malaria, which is more consistent with our findings (44). Interestingly, six out of the eight upregulated *Pf*EMP1 proteins identified in our samples contained an EPCR-binding CIDRα1 domain. Moreover, correlation analysis showed that the abundance of some of these proteins, including IGHvar09 and DD2var40, was positively associated with parasite adhesion to HBEC-5i cells, but not with adhesion to CHO-ICAM-1 cells. These findings suggest that parasites from CM patients may preferentially express *Pf*EMP1 variants associated with EPCR-mediated adhesion rather than ICAM-1-mediated adhesion. Overall, changes in *Pf*EMP1 abundance after normalization according to the estimated trophozoite/schizont proportions were limited, with only minor changes in abundance ratios. From a statistical perspective, significance was lost for only two *Pf*EMP1 proteins, among the least well-characterized candidates in the analysis (Supplemental Data 6b). This suggests that stage-based normalization has a stronger impact on well-characterized proteins from the global parasite proteome, whereas its effect is more limited for *Pf*EMP1 proteins, which are characterized by a higher proportion of missing values.

A limitation of our method is the low sensitivity of *Pf*EMP1 identification by LC-MS/MS. Indeed, this proteomic method only considers the intensity of a limited number of peptides that match *Pf*EMP1 proteins, and not the whole sequence. Therefore, the results are less representative of the full diversity of *var* genes than transcriptomic analyses. Analysis of *Pf*EMP1 domains revealed a significantly higher abundance of four domains in CM cases compared to UM: NTS, DBLα1.2, CIDRα1.1, and DBLδ1. Since the NTS domain is relatively common among all *Pf*EMP1 proteins, its increased abundance in CM cases may indicate a higher total amount of *Pf*EMP1 in parasitized RBCs. Interestingly, CIDRα1.1 (DC8) was also found to be more abundant in CM cases, suggesting increased binding to the EPCR. These data may suggest that the DBLα1.2 and DBLδ1 domains are also associated with CM.

In a subsequent analysis, we used a protein database derived from parasite isolates from African patients (Varia). We observed differences in *Pf*EMP1 domains between querying VarDom and Varia, as expected due to genetic variability between laboratory strains and field isolates. However, some domains such as NTS and DBLδ1 were consistently found to be significantly more abundant in CM compared to UM in both databases. The increase of these domains at the protein level supports the hypothesis of a global upregulation of *var* gene expression in patients with CM compared to UM, as previously described (26). Varia also drastically improves the number of matching peptides compared to the VarDom database, allowing more *Pf*EMP1 domains to be identified, and suggesting more robust quantitative data. However, the main obstacle to the systematic use of Varia is the redundancy of genomic *var* sequences, which in proteomics makes it difficult to discriminate very similar amino acid sequences based on a small number of identified peptides.

In conclusion, we implemented methodological improvements, particularly the use of DIA, which significantly improved the quality of our differential protein expression analysis compared to our previous study using data-dependent acquisition (29). These improvements allowed us to present new findings from the proteome analysis of parasitized RBCs. Consistently with our previous hypothesis, these data appear to indicate, albeit preliminarily, that *P. falciparum* may preferentially infect erythroid precursors of CM patients. We also demonstrated a higher abundance of several VSAs such as RIFIN and STEVOR as well as PHIST family proteins in CM compared to UM. Furthermore, *Pf*EMP1 proteins containing DC8 and DC13 that are associated with CM were more abundant in CM cases compared to UM, providing the first protein-level confirmation of numerous previous transcriptomic analyses of field isolates. Taken together, these proteomic data on *Pf*EMP1 suggest an increased parasite adhesion mechanism in CM compared to UM, consistent with recent *in vitro* (25, 45) and transcriptomic studies (26, 46).

## Data Availability

Proteomic acquired raw data are available on ProteomeXchange *via* PRIDE (https://www.ebi.ac.uk/pride): Project accession: PXD061411.

Reviewer account details: Username: reviewer_pxd061411@ebi.ac.uk, Password: Kt2VogHKSIOc.

## Supplemental data

This article contains supplemental data (47).

## Declaration of AI and AI-Assisted Technologies in the Writing Process

During the preparation of this article, the authors used ChatGPT (OpenAI) to assist with French to English translation and to improve the clarity, grammar, and phrasing of the English text. All AI-assisted text was subsequently reviewed and edited by the authors, who take full responsibility for the final content. We generated figure supports for the graphical abstract, and used some Biorender graphical design.

## Conflict of Interest

The authors declare no competing interests.

## Supplemental data

Supplemental Data 1 String enrichment of human proteins. Enrichment analysis of all human proteins using String online tool (https://string-db.org/).

Supplemental Data 2 Human differential of expression. Table of human proteins identified and quantified following the DIA-NN analysis B. Only human proteins were kept, DIA-NN protein abundance values for each sample (left side of the table) were log2-transformed, filtered based on valid values (≥70% present in at least one group), and subsequently analyzed using a Student’s *t* test with Benjamini–Hochberg correction. A “+” symbol in the AP or AQ columns indicates that the protein is significantly deregulated (q-value <0.05).

Supplemental Data 3 String humans q-val. Over-Representation Analysis (ORA) of the differentially expressed human proteins (n = 61) using String online tool (https://string-db.org/).

Supplemental Data 4 *P. falciparum* differential of expression. Table of *P. falciparum* proteins identified and quantified following the DIA-NN analysis B. Only *P. falciparum* proteins were kept, DIA-NN protein abundance values for each sample (left side of the table) were log2-transformed, filtered for valid values (≥70% present in at least one group), and subsequently analyzed using a Student’s *t* test without Benjamini–Hochberg correction. A “+” symbol in the AP or AQ columns indicates that the protein is significantly deregulated (AP: *p* value <0.01; AQ: q-value <0.05). Notably, only six proteins passed the significance threshold after Benjamini–Hochberg correction; these proteins are highlighted in red in the table.

Supplemental Data 4b The same data processing workflow as in Supplemental Data 4 was applied, with LFQ values corrected for parasite stage proportions (trophozoites/schizonts).

Supplemental Data 5 Comparison of parasite protein differential abundance before and after parasite stage normalization. This table reports the statistical parameters obtained from the two analyses of parasite proteins before and after normalization of LFQ intensities according to the estimated trophozoite and schizont proportions in each sample. “Original” refers to the analysis performed without stage normalization, whereas “Stage-normalized” refers to the analysis performed after normalization. The “status” columns indicate whether protein significance was gained, lost, or maintained after stage normalization.

Supplemental Data 6 VarDom differential of expression. Table of protein identification and quantification obtained after DIA-NN analysis A focusing on the *Pf*EMP1 proteins of *P. falciparum* based on VarDom database. DIA-NN protein abundance values for each sample (left side of the table) were log2-transformed, no protein filtering was applied for FL-*Pf*EMP1 and for *Pf*EMP1 domains only domains with at least 2 valid values in at least 1 group were kept. Then a Student’s *t* test without Benjamini–Hochberg correction was done. A “+” symbol in the “U” column indicates that the protein is significantly deregulated (*p* value <0.05). The tab called “VarDom_*Pf*EMP1_FL” displayed full-length *Pf*EMP1 proteins and the second (VarDom_*Pf*EMP1_Domains) displayed *Pf*EMP1 truncated domains.

Supplemental Data 6b The same data processing workflow as in Supplemental Data 6 was applied, with LFQ values corrected for parasite stage proportions (trophozoites/schizonts).

Supplemental Data 7 Varia differential of expression. Table of protein identification and quantification obtained after DIA-NN analysis C focusing on the *Pf*EMP1 domains of *P. falciparum* based on Varia database. DIA-NN protein abundance values for each sample (left side of the table) were log2-transformed, filtered for at least 40% valid values in at least one group and subsequently analyzed using a Student’s *t* test without Benjamini–Hochberg correction. A “+” symbol in the “T” column indicates that the protein is significantly deregulated (*p* value <0.05).

Supplemental Data 8 Varia_Mean differential of expression. Table summarizing protein identification and quantification obtained from DIA-NN and Perseus analyses, focusing on *P. falciparum Pf*EMP1 domains annotated using the Varia database. Abundances of identical domains from different *Pf*EMP1 proteins were averaged and reported as a single domain expression value. DIA-NN protein abundance values for each sample (left side of the table) were log2-transformed, filtered for at least 40% valid values in at least one group and subsequently analyzed using a Student’s *t* test without Benjamini–Hochberg correction. A “+” symbol in the “T” column indicates that the protein is significantly deregulated (*p* value <0.05).

Supplemental Data 9 Varia peptides quantification. Table comparing the total number of peptides identified using the Varia and VarDom databases. Notably, 29 domains were identified only with the Varia database and were not detected with VarDom.

Supplemental Data 10 Characterization of peptide identifications. One table is provided for each analysis (VarDom Protein, VarDom Domain, and Varia), reporting the number of unique peptides per protein group, the total number of peptides identified per protein, and the total number of peptides identified per sample for each given protein.

Supplemental Methods

Supplemental Figure 1 **Qualitative and quantitative assessment of the data from sample CM10.***A*, principal component analysis of Z-scored protein abundances, including proteins with at least 70% valid values in at least one group (note that CM10 is clearly separated from the other samples along the first principal component). *B*, ingenuity pathway analysis (IPA) exploration of specific terms from Gene Ontology for human proteins. *C*, analysis of CM10 quantitative values among all other CM samples. *a*, scatter plot analysis of CM10 as compared with other CM samples, green proteins were selected because they are always more abundant in CM 10 than other samples. *b*, Pearson correlation between CM 10 and other CM samples. *c*, Venn diagram comparing the 203 selected green proteins (« atypical ») with IPA specific list for erythrocyte, leukocyte, and neutrophil.

Supplemental Figure 2 **Descriptive analysis of the global proteome.***A*, number of proteins identified per sample (*up*) and percentage of missing values per sample (*down*). *B*, Pearson’s correlation heatmap between samples (*left*) and Boxplot (*right*). *C*, principal component analysis (PCA) of the global proteome, including only proteins with 100% valid values in all group.

Supplemental Figure 3 **Parasite proteome analysis.***A*, Venn diagram of the parasite proteins identified in *P. falciparum*-infected erythrocyte from NeuroCM cohort. *B*, principal component analysis of the parasite protein quantified by LC-MS/MS, including only proteins with 70% valid values in at least one group and imputed with Gaussian distribution with Perseus tool.

Supplemental Figure 4 **Stage normalization effect on quantitative data.***A*, principal component analysis of parasite proteins (DIA-NN analysis B), including only proteins with 100% valid values in all group. *B*, principal component analysis of parasite proteins (DIA-NN analysis B), including only proteins with 100% valid values in all group after correction for trophozoite/schizont normalization.
