# Supplementary material for: Global Proteomic Profiling of Cerebral Malaria-Infected Erythrocytes Reveals a Specific PfEMP1 Expression Pattern
Source: Mol Cell Proteomics. 2026 Sep 10;25(10):101659. doi: 10.1016/j.mcpro.2026.101659 (PMC13682175; doi:10.1016/j.mcpro.2026.101659)

Supplemental figure 1

CM10 Sample analysis

Supplemental Figure 1 A:

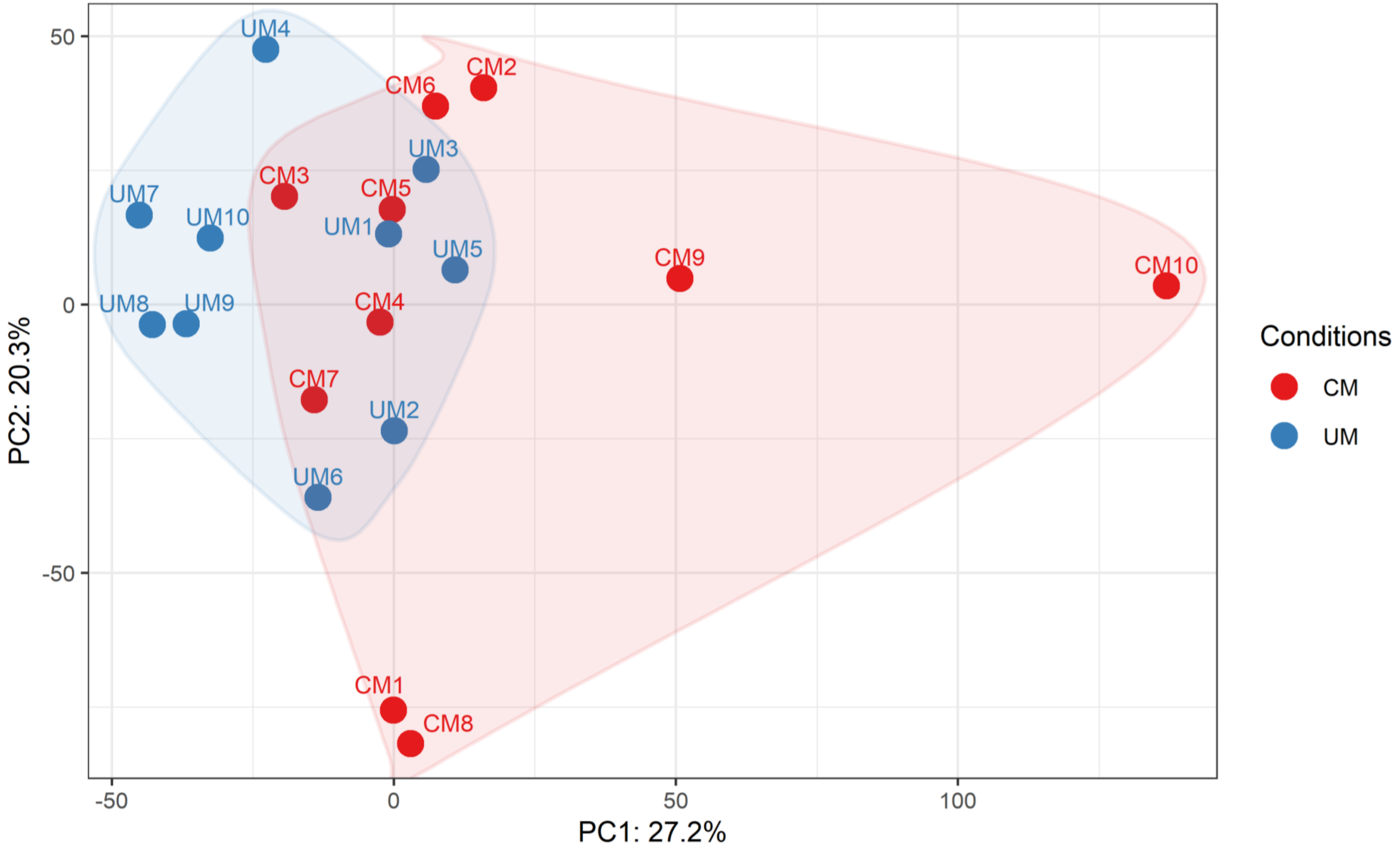

Supplemental Figure 1 B:

Plasma

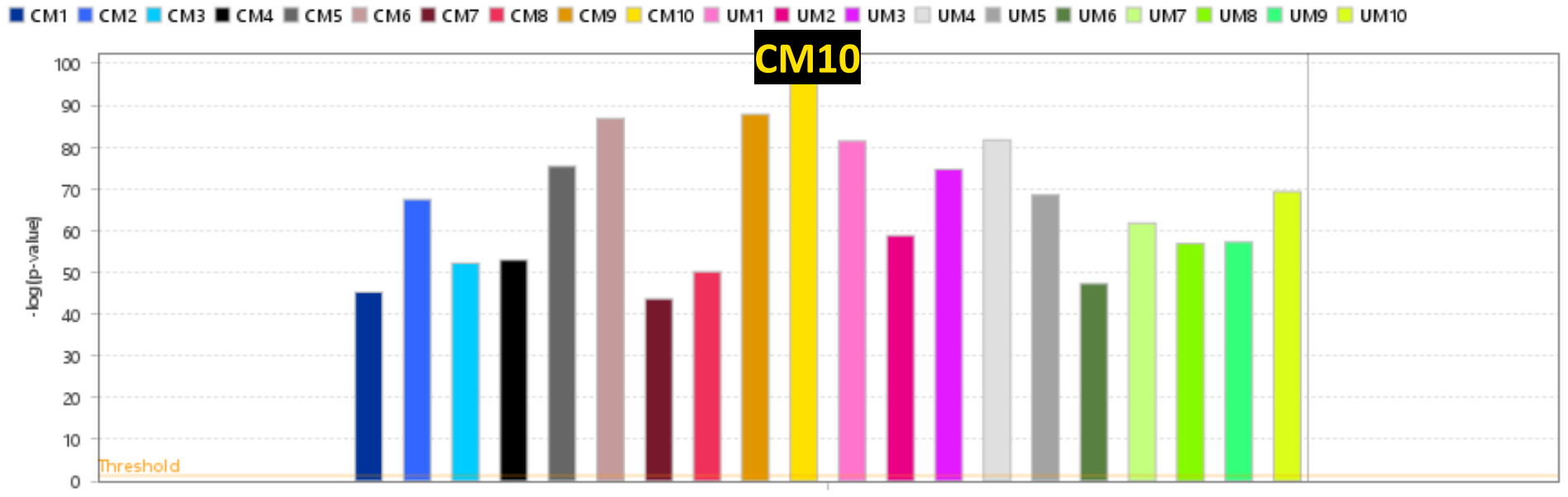

RNA Binding

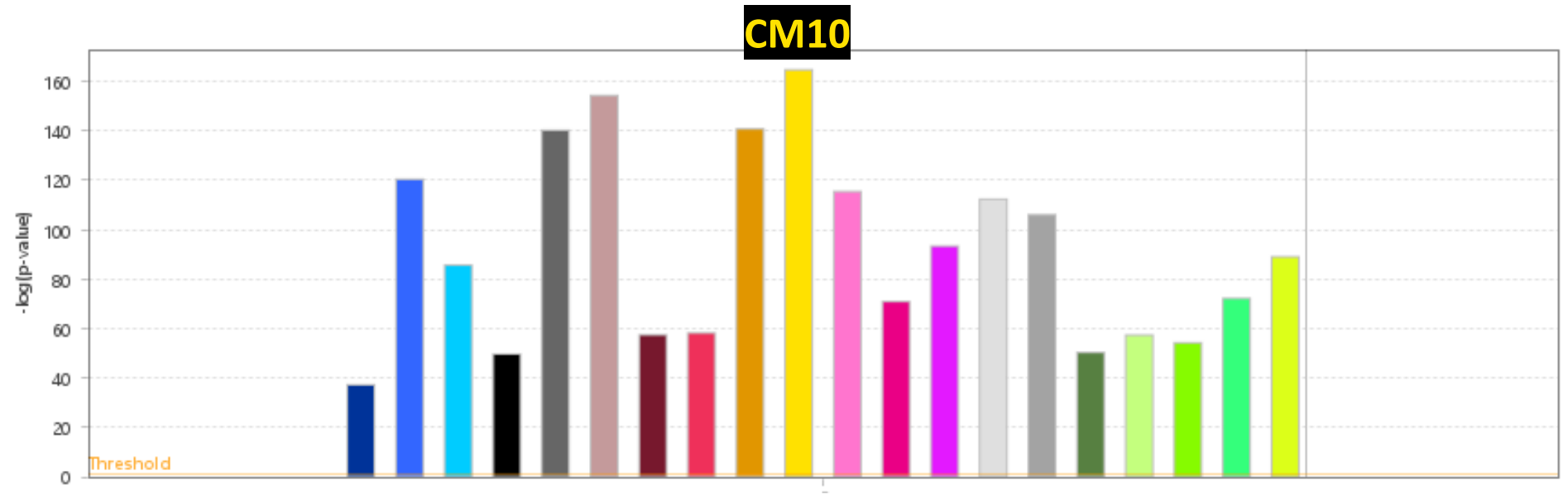

Erythrocyte

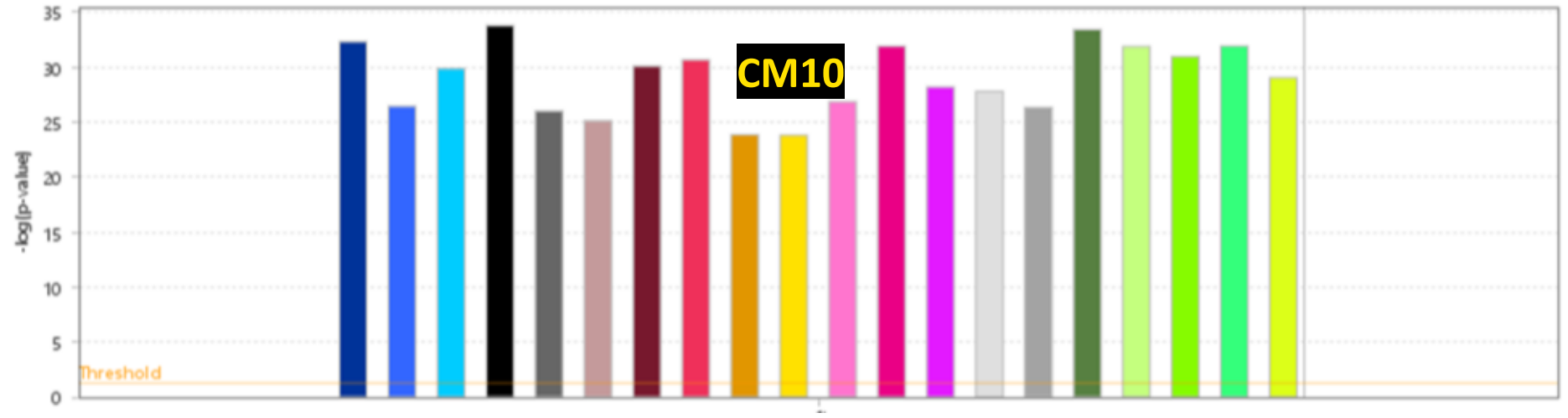

Leucocytes

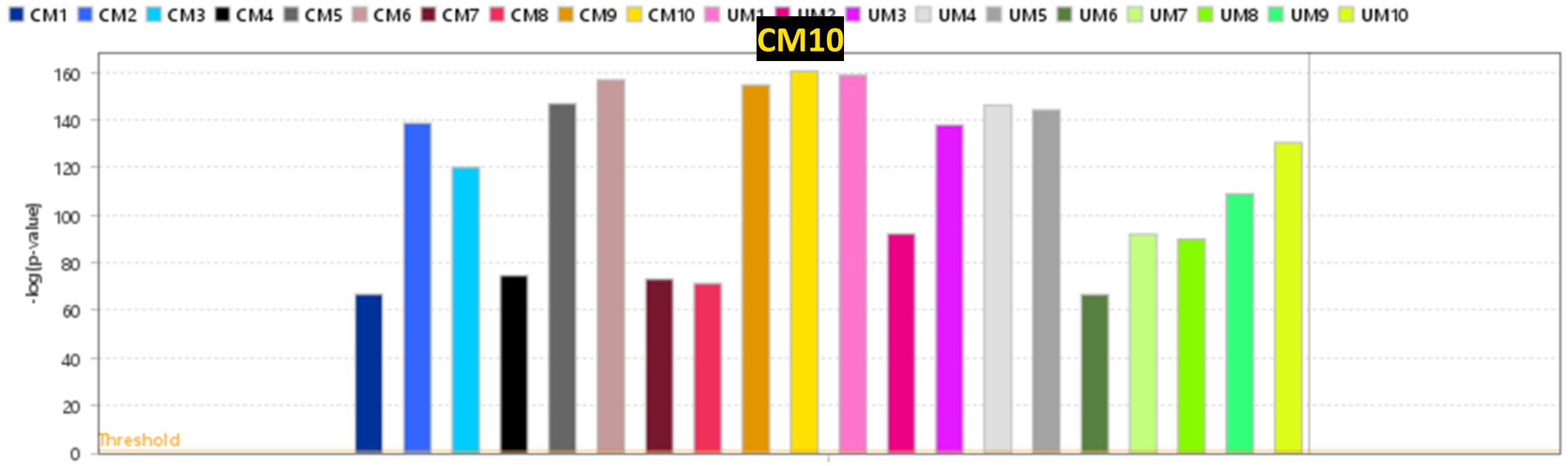

Neutrophils

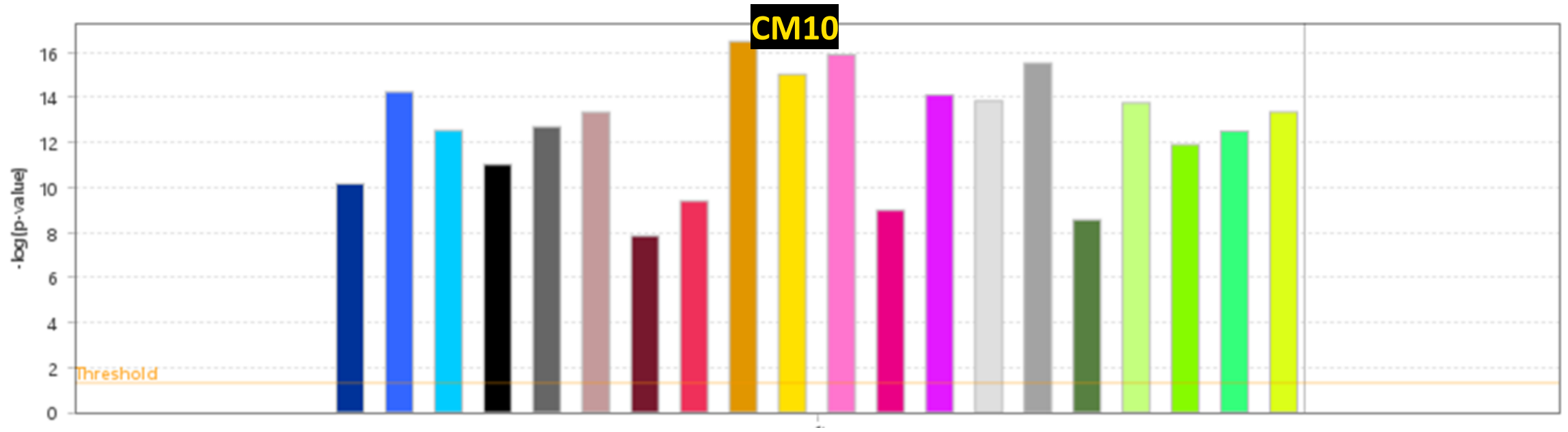

Supplemental Figure 1 C

a.

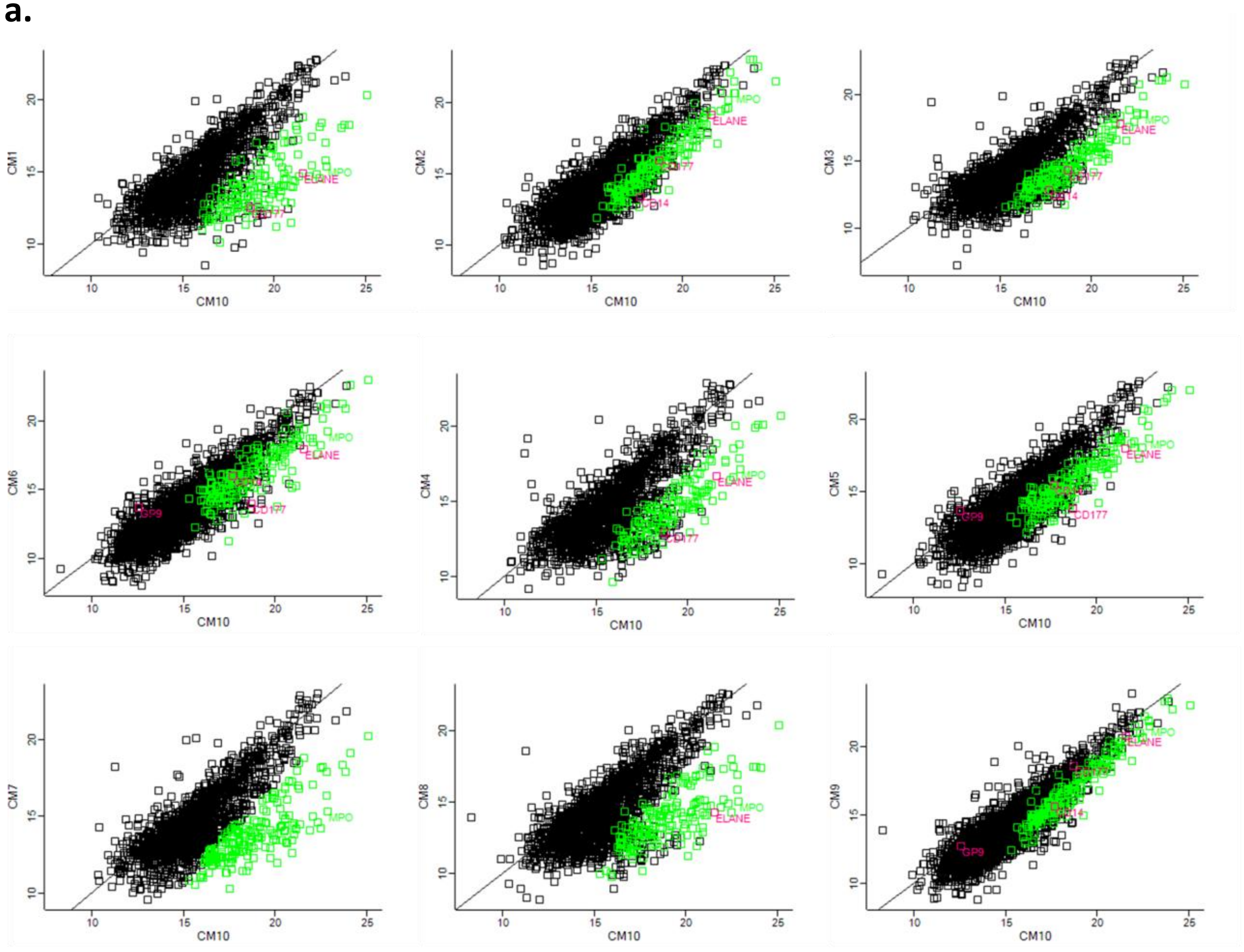

b.

**Sample Y**	**Sample X**	**R-squared**
CM1	CM10	**0,454201**
CM2	CM10	**0,695034**
CM3	CM10	**0,53038**
CM4	CM10	**0,470431**
CM5	CM10	**0,683955**
CM6	CM10	**0,764122**
CM7	CM10	**0,475873**
CM8	CM10	**0,435834**
CM9	CM10	**0,803076**

c.

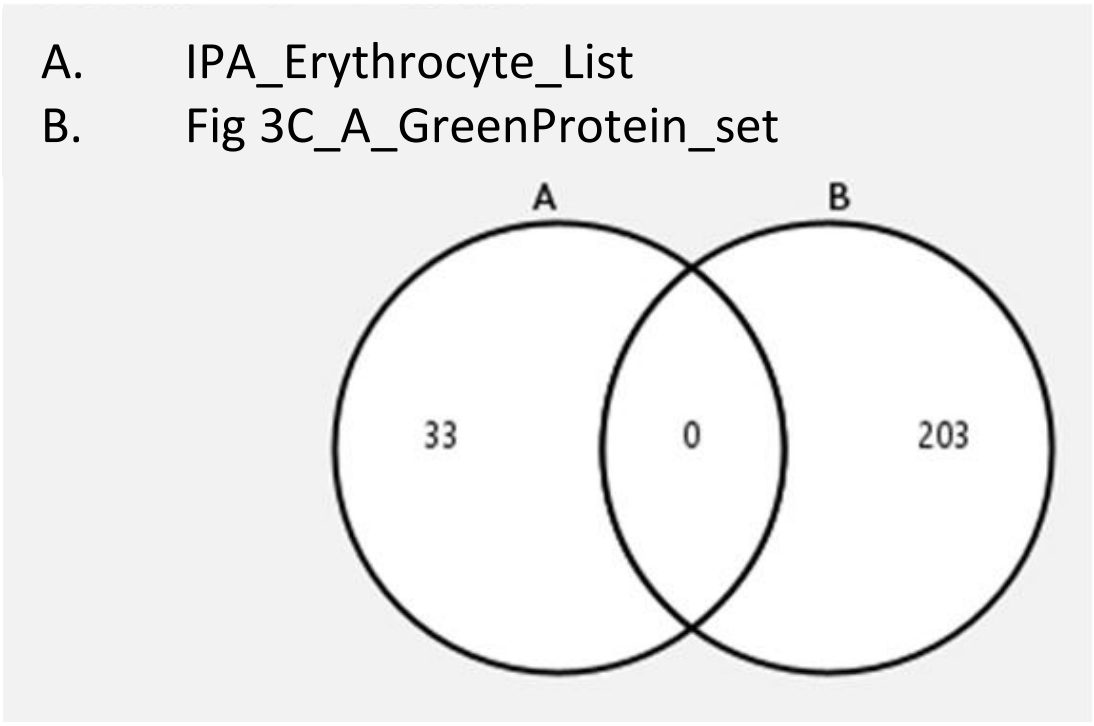

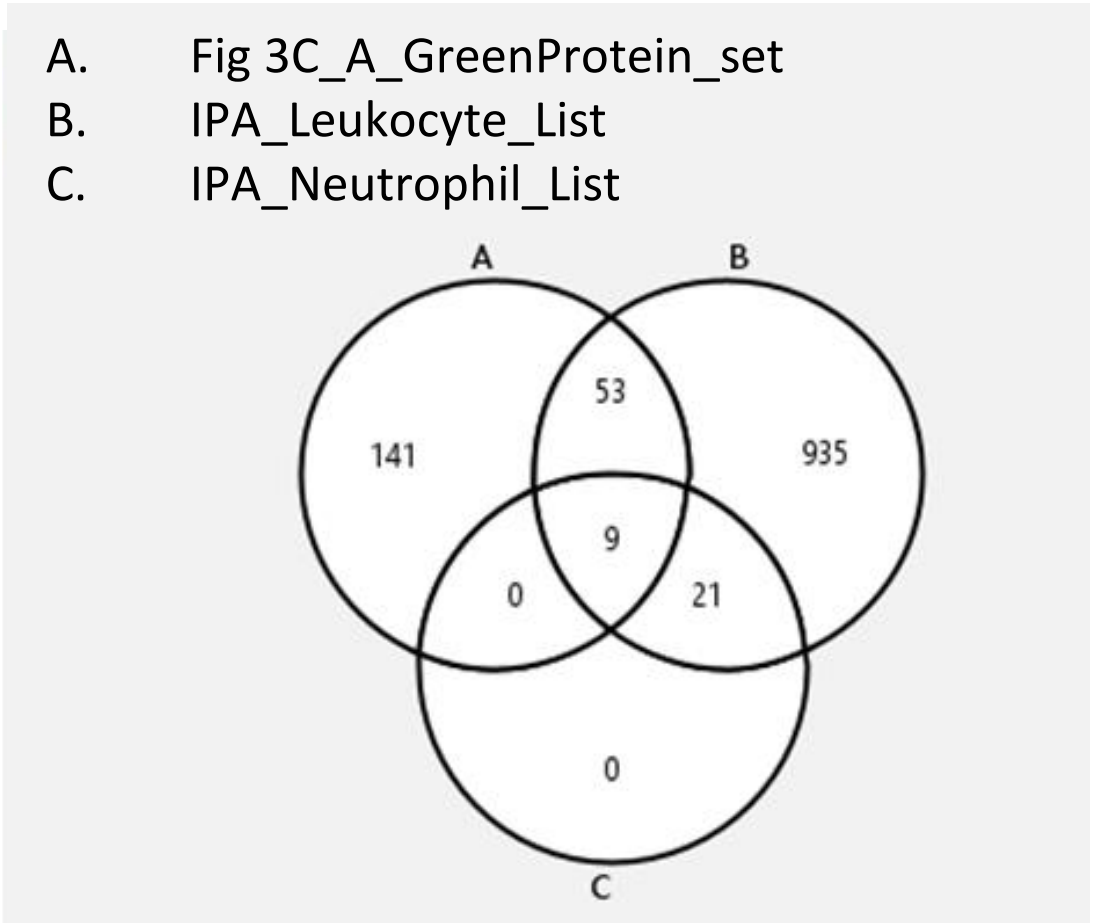

Supplement: Supplementary file 1 [file mmc14.pdf]
