# Supplementary material for: Global Proteomic Profiling of Cerebral Malaria-Infected Erythrocytes Reveals a Specific PfEMP1 Expression Pattern
Source: Mol Cell Proteomics. 2026 Sep 10;25(10):101659. doi: 10.1016/j.mcpro.2026.101659 (PMC13682175; doi:10.1016/j.mcpro.2026.101659)
Supplement: Supplementary file 2 [file mmc15.pdf]

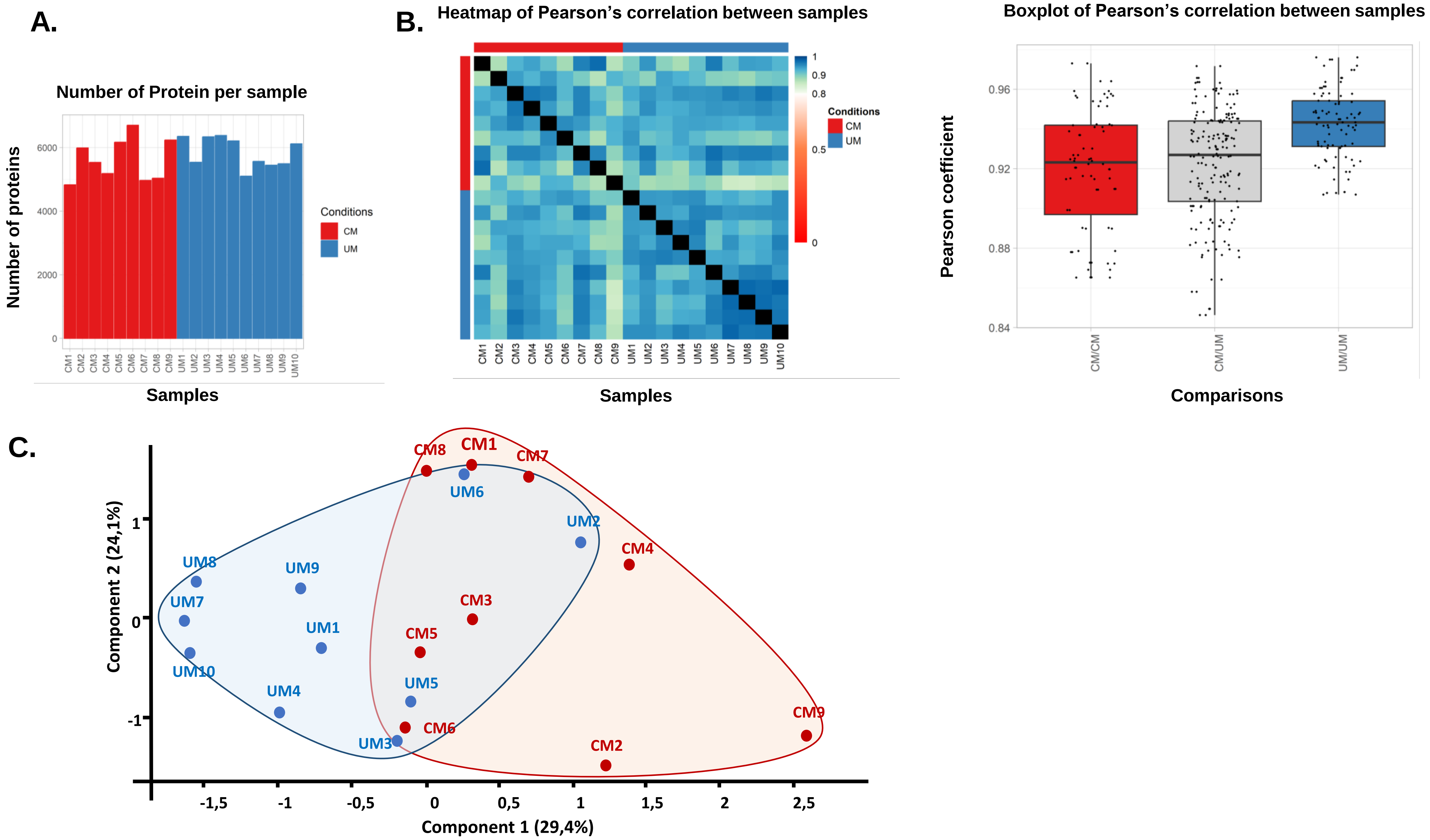
A.
Number of Protein per sample
Number of proteins
0
2000
4000
6000
Conditions
CM
UM
CM1 CM2 CM3 CM4 CM5 CM6 CM7 CM8 CM9 UM1 UM2 UM3 UM4 UM5 UM6 UM7 UM8 UM9 UM10
Samples
B.
Heatmap of Pearson's correlation between samples
1
0.9
0.8
0.5
0
Conditions
CM
UM
CM1 CM2 CM3 CM4 CM5 CM6 CM7 CM8 CM9 UM1 UM2 UM3 UM4 UM5 UM6 UM7 UM8 UM9 UM10
Samples
Boxplot of Pearson's correlation between samples
Pearson coefficient
0.84
0.88
0.92
0.96
CM/CM
CM/UM
UM/UM
Comparisons
C.
Component 2 (24,1%)
Component 1 (29,4%)
1
0
-1
-1,5
-1
-0,5
0
0,5
1
1,5
2
2,5
CM1 CM2 CM3 CM4 CM5 CM6 CM7 CM8 CM9
UM1 UM2 UM3 UM4 UM5 UM6 UM7 UM8 UM9 UM10
