# Supplementary material for: Global Proteomic Profiling of Cerebral Malaria-Infected Erythrocytes Reveals a Specific PfEMP1 Expression Pattern
Source: Mol Cell Proteomics. 2026 Sep 10;25(10):101659. doi: 10.1016/j.mcpro.2026.101659 (PMC13682175; doi:10.1016/j.mcpro.2026.101659)

A. **Parasite proteins**

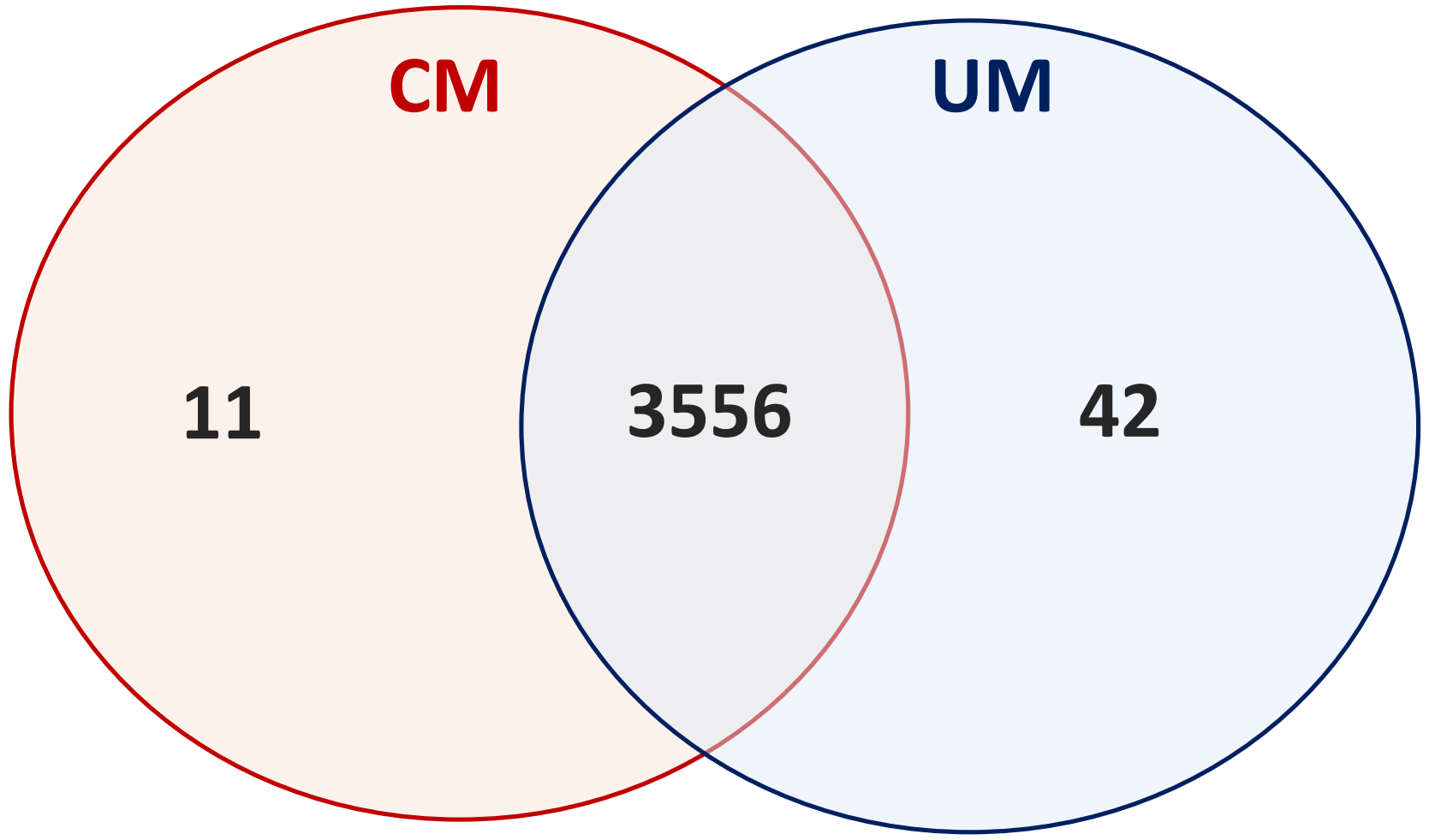

B. **Parasite proteins**

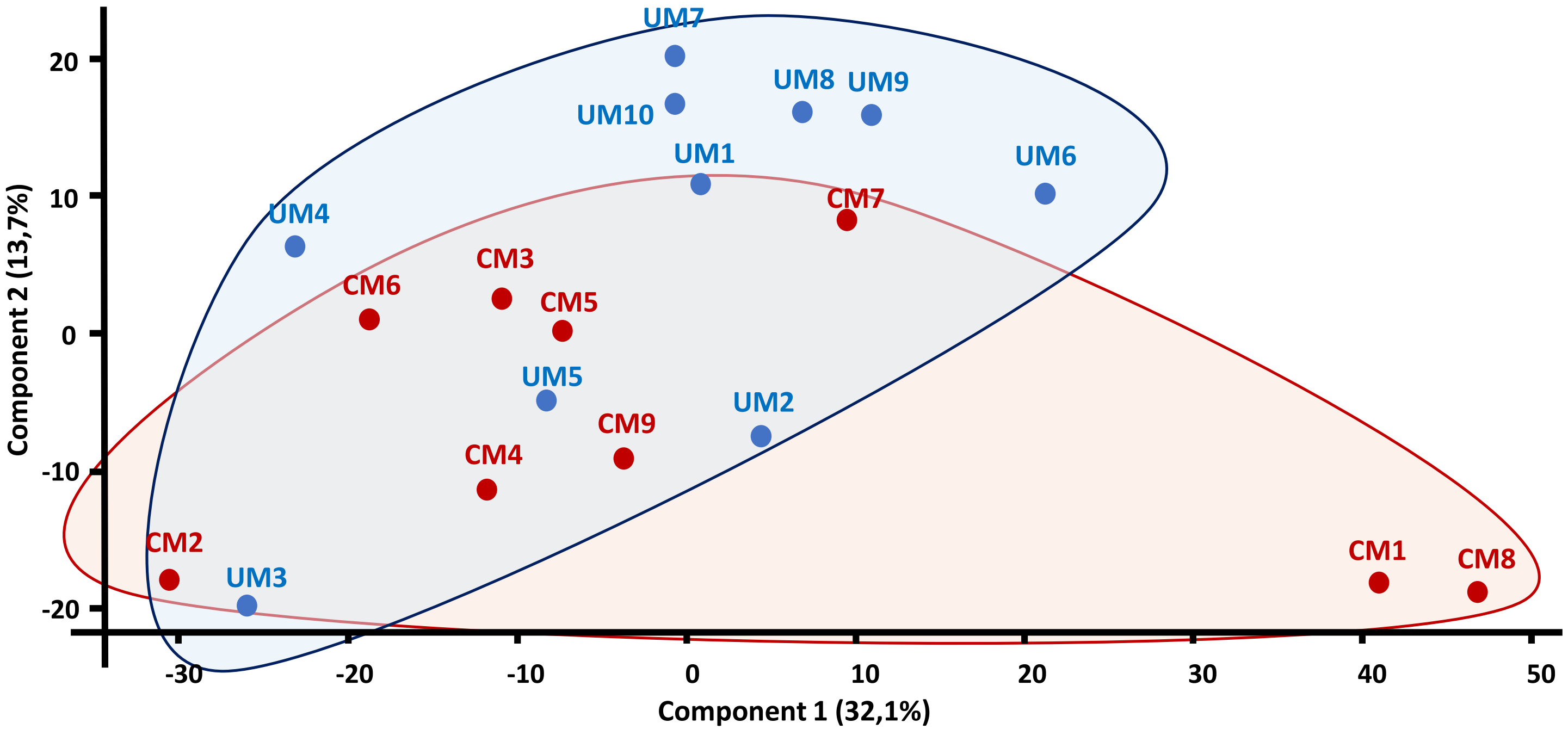

Supplement: Supplementary file 3 [file mmc16.pdf]
