# Supplementary material for: Global Proteomic Profiling of Cerebral Malaria-Infected Erythrocytes Reveals a Specific PfEMP1 Expression Pattern
Source: Mol Cell Proteomics. 2026 Sep 10;25(10):101659. doi: 10.1016/j.mcpro.2026.101659 (PMC13682175; doi:10.1016/j.mcpro.2026.101659)
Supplement: Supplementary file 4 [file mmc17.pdf]

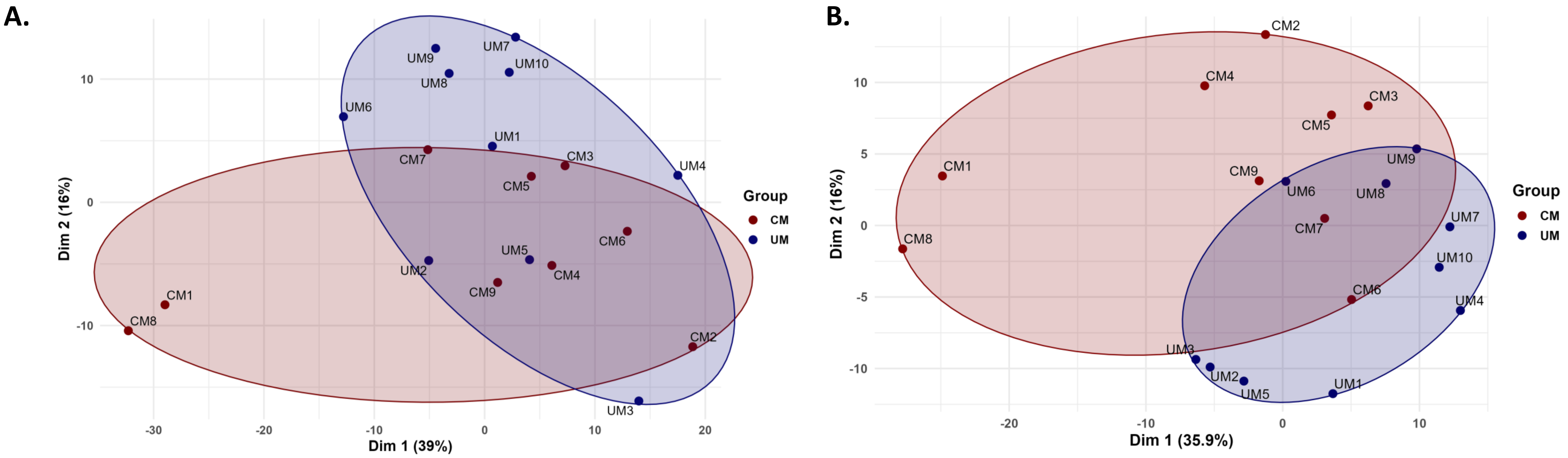
A.
UM9
UM7
UM10
UM8
UM6
UM1
CM7
CM3
UM4
CM5
CM6
UM5
UM2
CM4
CM9
CM1
CM8
CM2
UM3
Group
CM
UM
10
0
-10
-30
-20
-10
0
10
20
Dim 1 (39%)
Dim 2 (16%)
B.
CM2
CM4
CM3
CM5
CM1
CM9
UM9
UM6
UM8
CM7
UM7
CM8
UM10
CM6
UM4
UM3
UM2
UM5
UM1
Group
CM
UM
10
5
0
-5
-10
-20
-10
0
10
Dim 1 (35.9%)
Dim 2 (16%)
