# Supplementary material for: Global Proteomic Profiling of Cerebral Malaria-Infected Erythrocytes Reveals a Specific PfEMP1 Expression Pattern
Source: Mol Cell Proteomics. 2026 Sep 10;25(10):101659. doi: 10.1016/j.mcpro.2026.101659 (PMC13682175; doi:10.1016/j.mcpro.2026.101659)
Supplement: Supplementary file 5 [file mmc13.docx]

**Supplemental Methods**

**Supplemental Table A : All DIA windows used to acquire the LC-MS/MS data.**

| #MS Type | Cycle Id | Start IM [1/K0] | End IM [1/K0] | Start Mass [m/z] | End Mass [m/z] | CE [eV] |
| --- | --- | --- | --- | --- | --- | --- |
| MS1 | 0 | - | - | - | - | - |
| PASEF | 1 | 1,3112 | 1,4192 | 1175 | 1200 | - |
| PASEF | 1 | 1,2013 | 1,3093 | 1025 | 1050 | - |
| PASEF | 1 | 1,0913 | 1,1993 | 875 | 900 | - |
| PASEF | 1 | 0,9814 | 1,0894 | 725 | 750 | - |
| PASEF | 1 | 0,8715 | 0,9795 | 575 | 600 | - |
| PASEF | 1 | 0,7615 | 0,8695 | 425 | 450 | - |
| PASEF | 1 | 0,6452 | 0,7532 | 400 | 425 | - |
| PASEF | 2 | 1,2929 | 1,4009 | 1150 | 1175 | - |
| PASEF | 2 | 1,1829 | 1,291 | 1000 | 1025 | - |
| PASEF | 2 | 1,073 | 1,181 | 850 | 875 | - |
| PASEF | 2 | 0,9631 | 1,0711 | 700 | 725 | - |
| PASEF | 2 | 0,8531 | 0,9612 | 550 | 575 | - |
| PASEF | 2 | 0,7432 | 0,8512 | 400 | 425 | - |
| PASEF | 3 | 1,2196 | 1,3276 | 1050 | 1075 | - |
| PASEF | 3 | 1,1097 | 1,2177 | 900 | 925 | - |
| PASEF | 3 | 0,9997 | 1,1077 | 750 | 775 | - |
| PASEF | 3 | 0,8898 | 0,9978 | 600 | 625 | - |
| PASEF | 3 | 0,7799 | 0,8879 | 450 | 475 | - |
| PASEF | 3 | 0,6635 | 0,7715 | 425 | 450 | - |
| PASEF | 4 | 1,2379 | 1,3459 | 1075 | 1100 | - |
| PASEF | 4 | 1,128 | 1,236 | 925 | 950 | - |
| PASEF | 4 | 1,018 | 1,1261 | 775 | 800 | - |
| PASEF | 4 | 0,9081 | 1,0161 | 625 | 650 | - |
| PASEF | 4 | 0,7982 | 0,9062 | 475 | 500 | - |
| PASEF | 4 | 0,6818 | 0,7899 | 450 | 475 | - |
| PASEF | 5 | 1,2562 | 1,3642 | 1100 | 1125 | - |
| PASEF | 5 | 1,1463 | 1,2543 | 950 | 975 | - |
| PASEF | 5 | 1,0364 | 1,1444 | 800 | 825 | - |
| PASEF | 5 | 0,9264 | 1,0344 | 650 | 675 | - |
| PASEF | 5 | 0,8165 | 0,9245 | 500 | 525 | - |
| PASEF | 5 | 0,7002 | 0,8082 | 475 | 500 | - |
| PASEF | 6 | 1,2746 | 1,3826 | 1125 | 1150 | - |
| PASEF | 6 | 1,1646 | 1,2726 | 975 | 1000 | - |
| PASEF | 6 | 1,0547 | 1,1627 | 825 | 850 | - |
| PASEF | 6 | 0,9448 | 1,0528 | 675 | 700 | - |
| PASEF | 6 | 0,8348 | 0,9428 | 525 | 550 | - |
| PASEF | 6 | 0,7185 | 0,8265 | 500 | 525 | - |
| PASEF | 7 | 1,1765 | 1,2846 | 1125 | 1150 | - |
| PASEF | 7 | 1,0666 | 1,1746 | 975 | 1000 | - |
| PASEF | 7 | 0,9567 | 1,0647 | 825 | 850 | - |
| PASEF | 7 | 0,8467 | 0,9548 | 675 | 700 | - |
| PASEF | 7 | 0,7368 | 0,8448 | 525 | 550 | - |
| PASEF | 8 | 1,1949 | 1,3029 | 1150 | 1175 | - |
| PASEF | 8 | 1,0849 | 1,1929 | 1000 | 1025 | - |
| PASEF | 8 | 0,975 | 1,083 | 850 | 875 | - |
| PASEF | 8 | 0,8651 | 0,9731 | 700 | 725 | - |
| PASEF | 8 | 0,7551 | 0,8631 | 550 | 575 | - |
| PASEF | 9 | 1,2132 | 1,3212 | 1175 | 1200 | - |
| PASEF | 9 | 1,1033 | 1,2113 | 1025 | 1050 | - |
| PASEF | 9 | 0,9933 | 1,1013 | 875 | 900 | - |
| PASEF | 9 | 0,8834 | 0,9914 | 725 | 750 | - |
| PASEF | 9 | 0,7735 | 0,8815 | 575 | 600 | - |
| PASEF | 10 | 1,1216 | 1,2296 | 1050 | 1075 | - |
| PASEF | 10 | 1,0116 | 1,1197 | 900 | 925 | - |
| PASEF | 10 | 0,9017 | 1,0097 | 750 | 775 | - |
| PASEF | 10 | 0,7918 | 0,8998 | 600 | 625 | - |
| PASEF | 11 | 1,1399 | 1,2479 | 1075 | 1100 | - |
| PASEF | 11 | 1,03 | 1,138 | 925 | 950 | - |
| PASEF | 11 | 0,92 | 1,028 | 775 | 800 | - |
| PASEF | 11 | 0,8101 | 0,9181 | 625 | 650 | - |
| PASEF | 12 | 1,1582 | 1,2662 | 1100 | 1125 | - |
| PASEF | 12 | 1,0483 | 1,1563 | 950 | 975 | - |
| PASEF | 12 | 0,9384 | 1,0464 | 800 | 825 | - |
| PASEF | 12 | 0,8284 | 0,9364 | 650 | 675 | - |

**Supplemental Figure 1 : DIA windows scheme**

**Parasite stage estimation and linear regression model**

Parasite stage composition was estimated from LC-MS/MS Log2 LFQ intensities using predefined trophozoite- and schizont-associated marker proteins coming from (Pease BN et al. J Proteome Res. 2013 Sep 6) and those already known to be associated to Schizont/Merozoïtes forms like MSP1, AMA1. For the 5 Trophozoites markers: PF3D7_1118200; PF3D7_0500800; PF3D7_1408000; PF3D7_1407800; PF3D7_1408100. And for Schizontes: PF3D7_1342600; PF3D7_1218500; PF3D7_0930300; PF3D7_1133400; PF3D7_0731500. For each marker protein, Log2 LFQ values were standardized across samples using a z-score transformation:

z_mj = (LFQ_mj − mean(LFQ_m)) / sd(LFQ_m)

where LFQ_mj is the Log2 LFQ intensity of marker m in sample j, and mean(LFQ_m) and sd(LFQ_m) are calculated across all matched samples. For each sample, trophozoite-like and schizont-like scores were then calculated as the mean z-score of the five trophozoite markers and the five schizont markers, respectively:

T_j = mean(z_trophozoite markers,j)

S_j = mean(z_schizont markers,j)

These scores were converted into relative stage contributions using an exponential normalization:

Trophozoite-like percentage_j = 100 × exp(T_j) / [exp(T_j) + exp(S_j)]

Schizont-like percentage_j = 100 × exp(S_j) / [exp(T_j) + exp(S_j)]

Stage-adjusted Log2 LFQ values were then computed using a per-protein linear model. For each protein i, observed Log2 LFQ intensity was modeled as a function of the selected stage covariate:

Log2LFQ_ij = β0_i + β1_i × Stage_j + ε_ij

where Stage_j corresponds to the estimated trophozoite-like percentage, schizont-like percentage, or a derived stage-balance variable for sample j. Adjusted values were calculated by removing the estimated linear stage effect while centering the data to the mean stage composition of the samples used for that protein:

Log2LFQ_adjusted,ij = Log2LFQ_observed,ij − β1_i × (Stage_j − mean(Stage))

Proteins with fewer than five valid LFQ values were not adjusted. This procedure was used as a stage-adjusted sensitivity analysis to reduce the influence of differences in parasite developmental stage composition on protein abundance comparisons.

**Supplemental Table B : Estimated trophozoite-schizont ratio in each sample**

| **Sample Name** | **%Tropho** | **%Schizont** |
| --- | --- | --- |
| **CM1** | 69,6914481 | 30,3085519 |
| **CM2** | 5,68096108 | 94,3190389 |
| **CM3** | 46,0186534 | 53,9813466 |
| **CM4** | 17,8379082 | 82,1620918 |
| **CM5** | 47,3337943 | 52,6662057 |
| **CM6** | 30,7798179 | 69,2201821 |
| **CM7** | 73,5990157 | 26,4009843 |
| **CM8** | 73,5474645 | 26,4525355 |
| **CM9** | 53,6773834 | 46,3226166 |
| **UM1** | 52,5998891 | 47,4001109 |
| **UM2** | 57,1422764 | 42,8577236 |
| **UM3** | 9,72707249 | 90,2729275 |
| **UM4** | 38,1314858 | 61,8685142 |
| **UM5** | 33,9310022 | 66,0689978 |
| **UM6** | 83,472809 | 16,527191 |
| **UM7** | 62,6396911 | 37,3603089 |
| **UM8** | 72,7192566 | 27,2807434 |
| **UM9** | 81,7624478 | 18,2375522 |
| **UM10** | 68,0341265 | 31,9658735 |
